# Gap of Research to Practice in IC/BPS: A Scientometric Study of Available Evidence

**DOI:** 10.5152/tud.2025.24086

**Published:** 2025-04-04

**Authors:** Helia Mostafaei, Hamidreza Ashayeri, Sayeh Afshar, Hadi Mostafaei, Tannaz Aghaei Badr, Hanieh Salehi-pourmehr, Sakineh Hajebrahimi

**Affiliations:** 1 Students Research Committee, Tabriz University of Medical Sciences, Tabriz, Iran; 2 Department of Urology, Comprehensive Cancer Center, Medical University of Vienna, Vienna, Austria; 3 Research Center for Evidence-Based Medicine, Iranian EBM Centre: A JBI Centre of Excellence, Tabriz University of Medical Sciences Faculty of Medicine, Tabriz, Iran; 4 Department of Urology, Tabriz University of Medical Sciences Faculty of Medicine, Tabriz, Iran

**Keywords:** Bibliometric, bladder pain syndrome, interstitial cystitis, scientometric study

## Abstract

Our main objective is to first characterize the current status of the research field of interstitial cystitis and bladder pain syndrome (IC/BPS). This is achieved by mapping, analyzing, and sub-analyzing the field with a scientometric approach, which provides insights into future research. In September 2023, the Scopus database was searched without restrictions on language and date. The following search query was used: Bladder* W/5 pain* W/3 syndrome* AND Interstitial* W/3 (Cystitis* OR Cystides) and the Scopus filters were used to exclude short surveys, book chapters, editorials, notes, and guidelines. The author, journal, institution data, keywords, average citation, countries, and sources were transformed using a Biblioshiny tool (A Shiny app for Bibliometrix), accounting for author spelling, institutional naming, and subgrouping variations. The search strategy yielded 1833 studies. The USA was the most cited country, with other countries showing significant growth in recent years. In terms of affiliations, TZU CHI University was the most relevant in this field (n = 154). The most relevant keywords were “female,” “interstitial cystitis,” “human,” “cystitis interstitial,” “male,” and “cystalgia.” The current topics in IC/BPS research are Genome-wide association and Cannabidiols. By understanding the past, scientific gaps, and the direction of the current research, researchers can aim for their research questions and conduct their research more organized. The scientific gaps of IC/BPS can be effectively understood by paying attention to the models used in this research, which are the results of the systemic analysis of the scientific products in this research field.

**Main Points**

- The research field of IC/BPS has significantly expanded since the year 2000, with the USA and China emerging as the most prolific and highly cited countries.
- Thematic analysis identifies “motor themes” representing well-researched clinical interventions (e.g., Pentosan Polysulfate, Botulinum Toxin A) and “declining themes” that may indicate a shift in research priorities (e.g., animal studies, basic protein expression).
- Despite the growth in research, the persistent lack of a known pathophysiology and definitive treatment for IC/BPS highlights a significant translational gap. The identification of “basic themes” (important topics with insufficient knowledge) suggests potential areas for future research focus to address this gap and advance clinical practice.

## Introduction

Interstitial cystitis bladder pain syndrome or IC/BPS is a hindering urological condition characterized by chronic pelvic pain and urinary symptoms.^1^ Interstitial cystitis and bladder pain syndrome have been found to have a relation to other comorbidities, like fibromyalgia, irritable bowel disease, and migraines.^2^ Studies have also focused on the risk factors of developing IC/BPS. Stimulatory food and drinks, history of hormonal treatments, and rectal diseases.^3^,^4^ Many phenomena (e.g., inflammation, pelvic floor muscle dysfunction) are proposed as contributing factors for IC/BPS symptoms,^5^,^6^ but the exact pathophysiology of IC/BPS remains unknown. Historically, IC/BPS was characterized by Hunner lesions presented in cystoscopy, but many patients with IC/BPS lack this finding.^7^ All the research done on IC/BPS shapes the perception of the disease. Still, there is no known pathophysiology and treatment for the condition, and the management focuses on reducing the risk factors and pain.^8^ Although there have been many advances in research and technology, there remains a substantial gap between the knowledge retained in research and laboratories and translating it into effective clinical practice for patients. Bibliometric analysis is a useful technique for analyzing publications in a particular research area and identifying trending topics in those areas.^9^ This analysis reveals the mapping and impact evaluation of journals, authors, institutions, countries, and articles in the focused research area. It helps researchers gain an overview of the past, present, and future direction of the research.^10^ It was decided to explore the historical development of research, mapping, and identifying important surveys on this subject, as well as the challenges and underlying factors contributing to this knowledge gap.

## Methods

We employed bibliometric methods to conduct a comprehensive analysis of the literature on IC/BPS. Given the evolving nature of the definition and term used for IC/BPS in recent years, this approach was inclusive, with no restriction on a specific definition, development of theoretical concepts, research sub-themes, or contributions made by specific authors, organizations, or nations/regions. This allowed us to encompass seminal manuscripts in the research field, providing a thorough understanding of the topic.

### Search Strategies

In September 2023, a search was conducted in the Scopus database without year or language limitation. The following query was entered: Bladder* W/5 pain* W/3 syndrome* AND Interstitial* W/3 (Cystitis* OR Cystides). The Scopus filters excluded sources like Short Surveys, book chapters, editorials, notes, and guidelines.

### Inclusion Criteria

The literature found on Scopus was meticulously screened based on their titles and abstracts. Only research that focused on IC/BPS and its various aspects was deemed relevant and included in this survey. The selection and categorization of the studies into Animal and Basic Sciences studies, diagnostic studies (biomarkers, gene expressions), therapeutic studies, and observational studies (etiology, risks, prevalence, or incidence) were carried out by 2 independent authors with extensive expertise in the field.

### Analysis

The Scopus analysis tool was utilized to identify articles, citations, countries, and authors. Institutions. To rank the main outcomes (countries, journals, institutions, and authors’ impact), the number of research articles and citations was used. The most relevant keywords and trending topics in the IC/PBS research field were also ranked by the number of articles in each topic and keywords. For the Bibliometrix analysis, the Biblioshiny tool (A Shiny app for Bibliometrix) was used to transform all the data, including authors, journals, institutions, time span, sources, keywords, countries, and average citations. While transferring data into the Biblioshiny tool, multiple variations of author name, subgroup, and institutional name spellings were considered.

## Results

**Figure 1.**
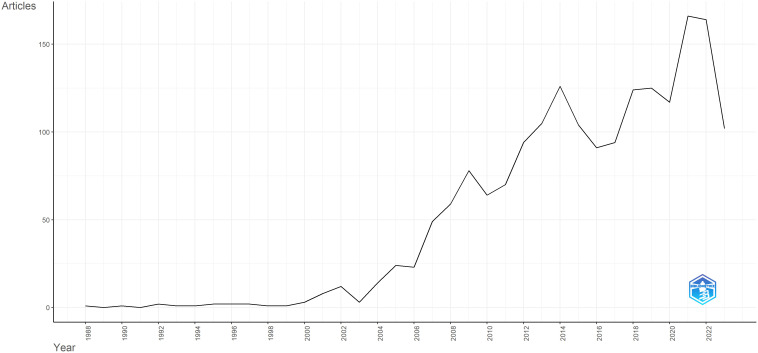
Annual scientific production.

**Figure 2.**
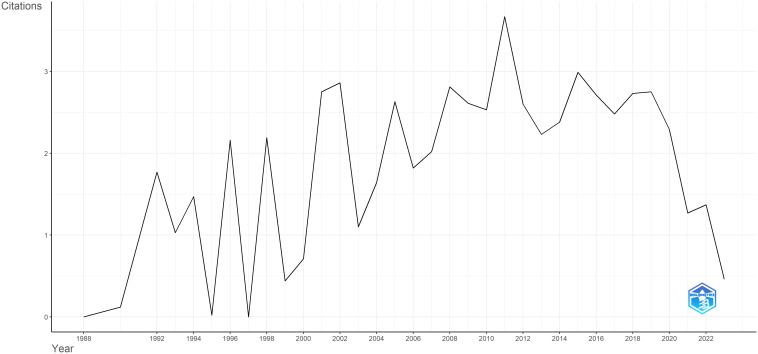
Average citation per year.

A total number of 1833 articles were found from the search, and the study selection was done by 2 professionals. The included studies have a time span of 1988 to 2023, and the annual scientific production represented in Figure 1 shows a great rise in scientific production after the year 2000. The average number of citations per document was 20.75, and Figure 2 shows the average article citation per year. Table 1 demonstrates the main scientometric information of the included studies.

### Collaboration Network

**Figure 3.**
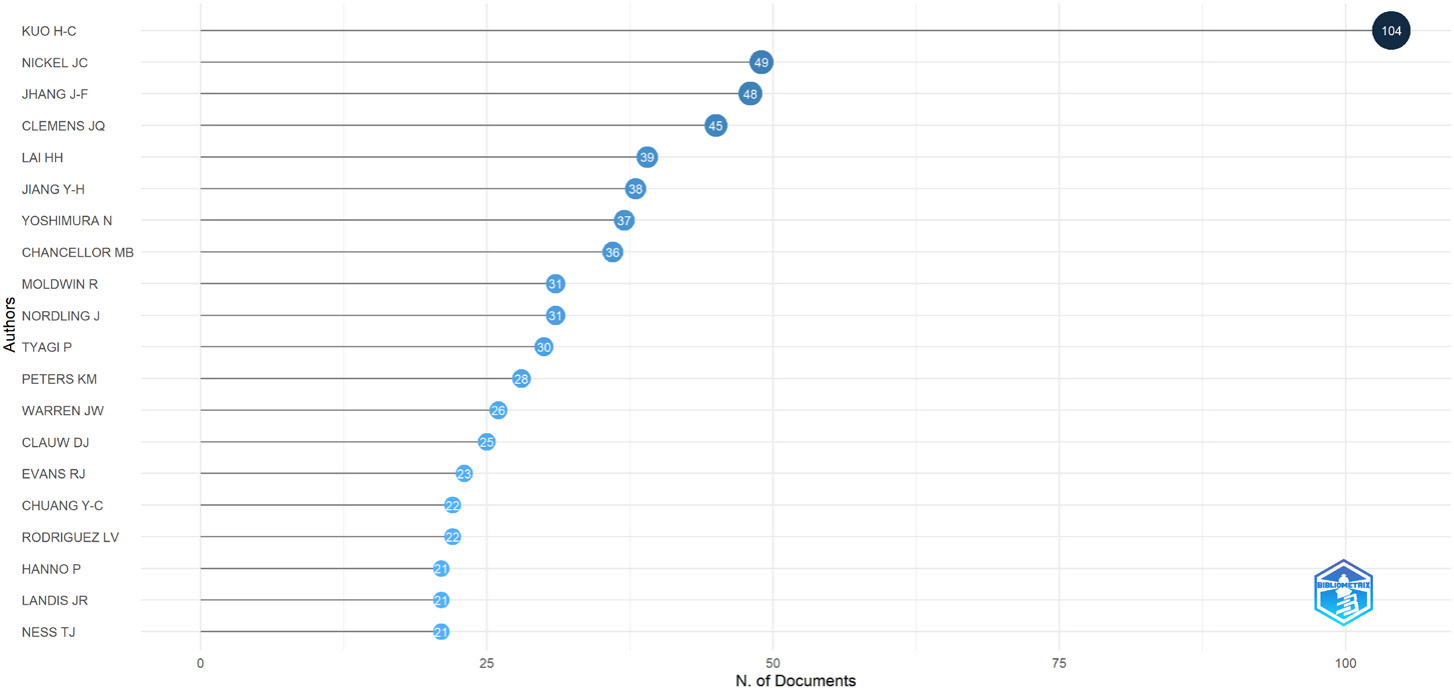
Most relevant authors.

**Figure 4.**
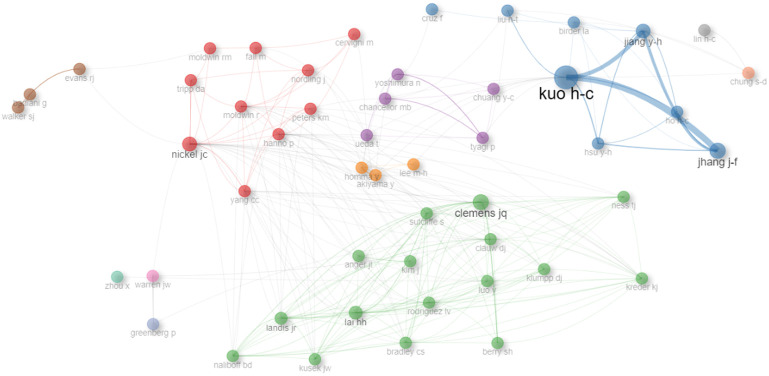
Authors collaboration network.

**Figure 5.**
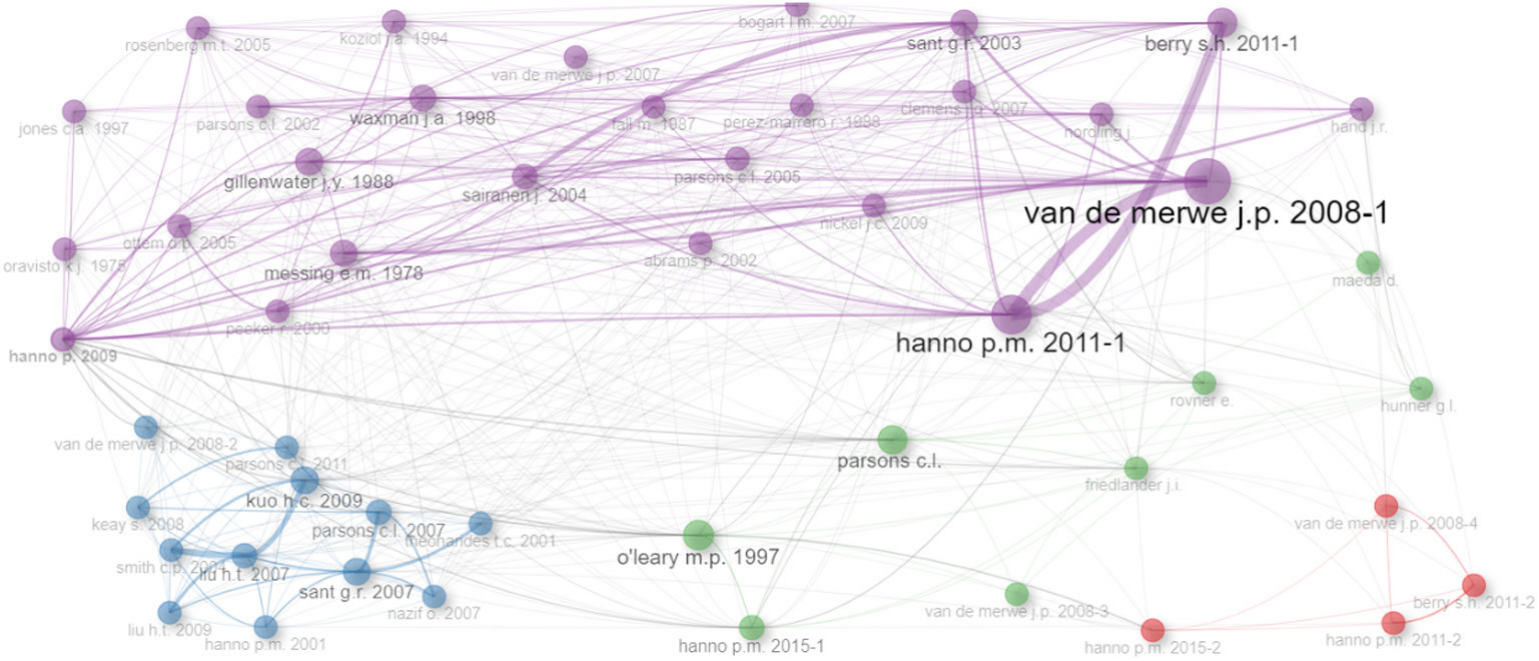
Co-citation network.

**Figure 6.**
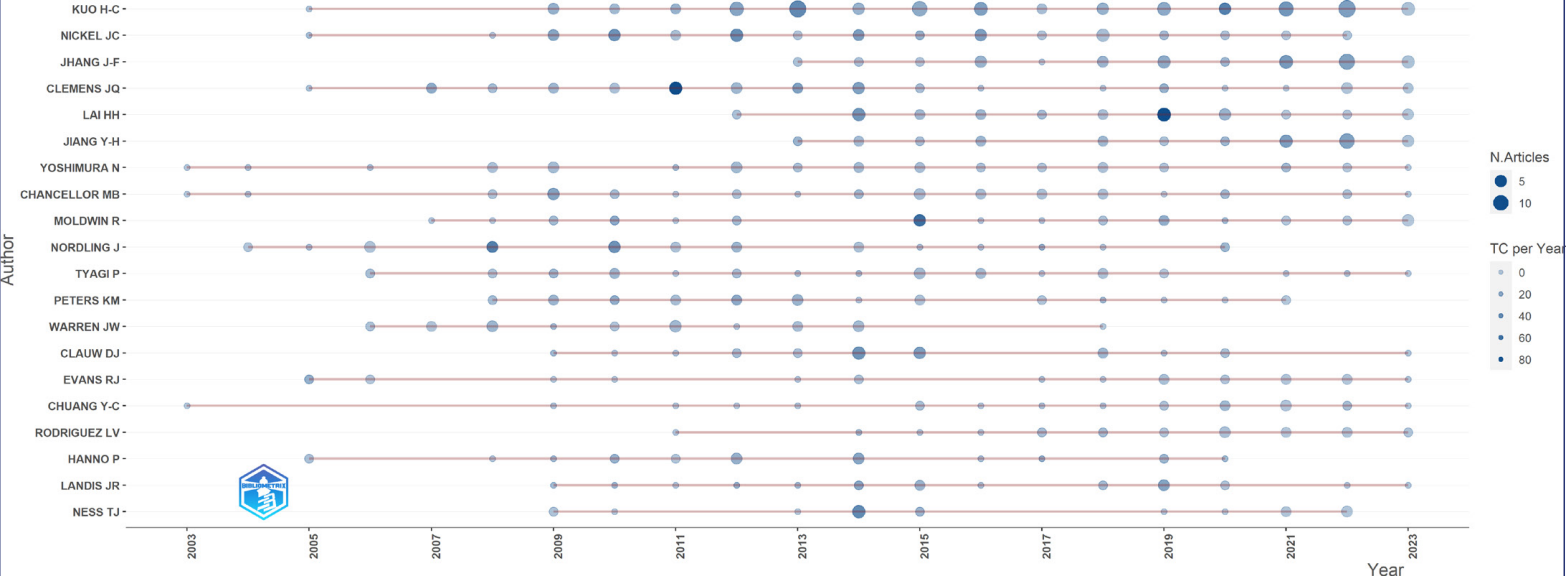
Authors’ production over times.

According to Figure 3, the most relevant authors, Kuo H.C. (number of documents: 104) and Nickel J,C, (number of documents: 49). Figure 4. demonstrates the authors with the most publications and the collaboration networks, with the size of each node indicating the number of publications and the thickness of the lines showing the intensity of collaboration. According to Figure 5, the co-citation network, Van de Merwe and Hanno P.M. were the authors with the most co-cited articles. Figure 6 is a representation of author production from 2003 to 2023.

**Figure 7.**
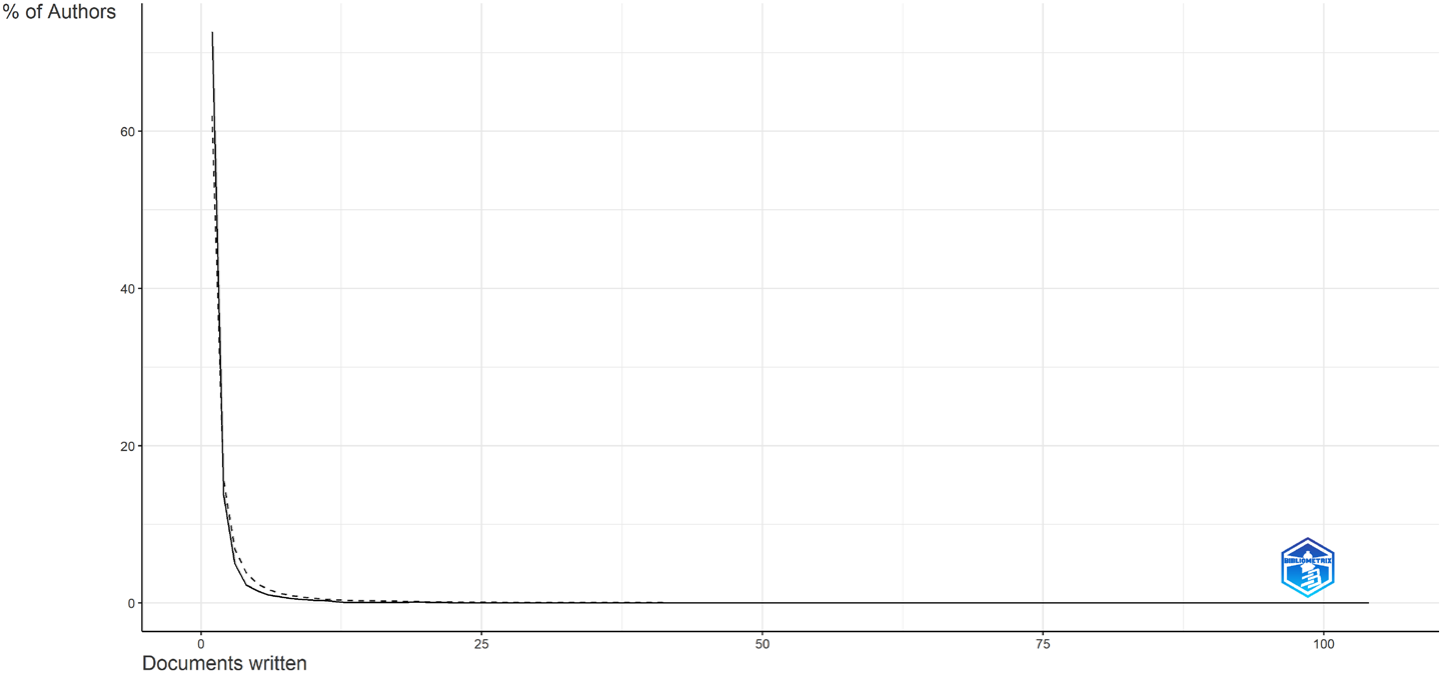
Authors’ production through Lotka’s law.

Lotka’s law measures productivity in scientific fields by comparing the number of authors contributing to the number of publications in a specific field.^11^ Figure 7 demonstrates Lotka’s law in the IC/BPS field, which shows a decrease in author contribution as the number of documents increases.

### Network of Nations, Areas, and Organizations

**Figure 8.**
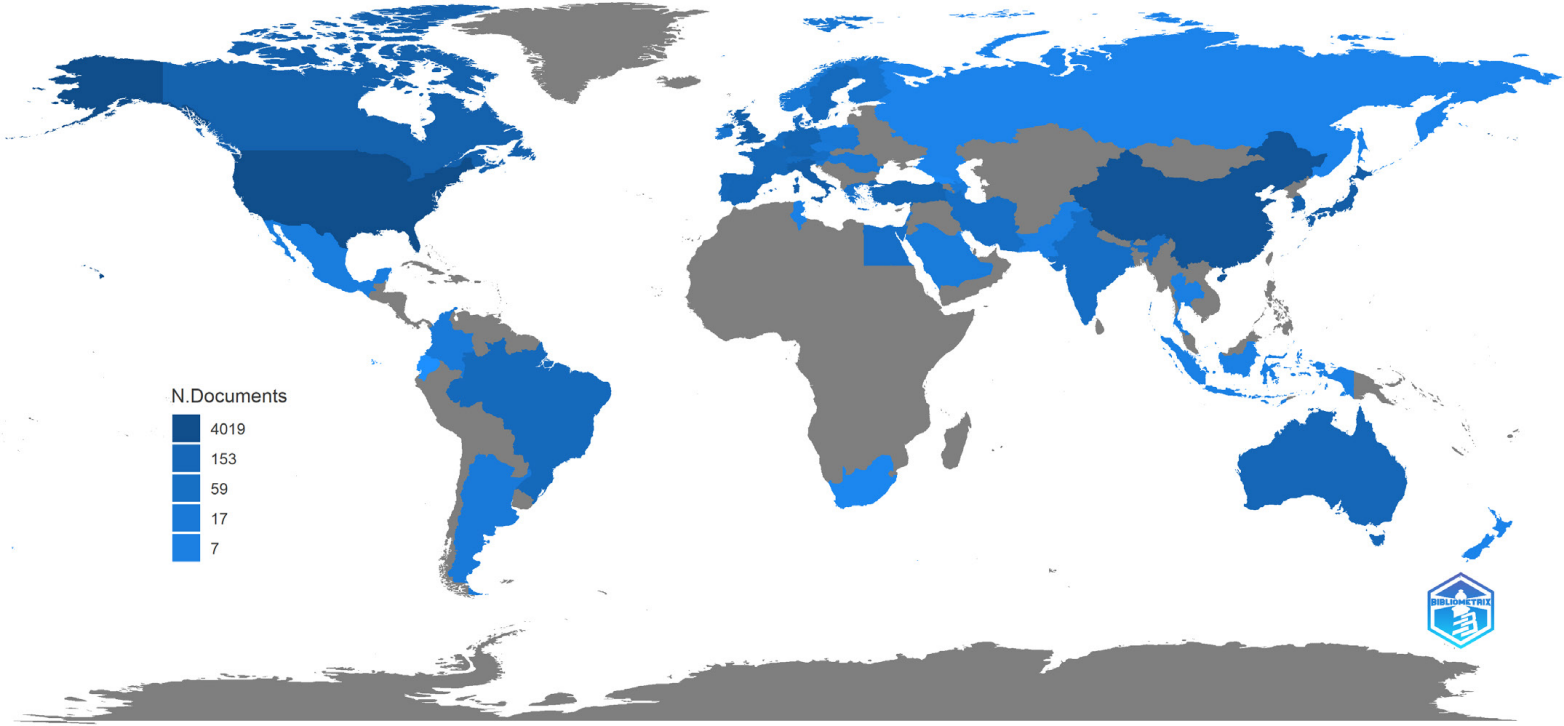
Country scientific production map.

**Figure 9.**
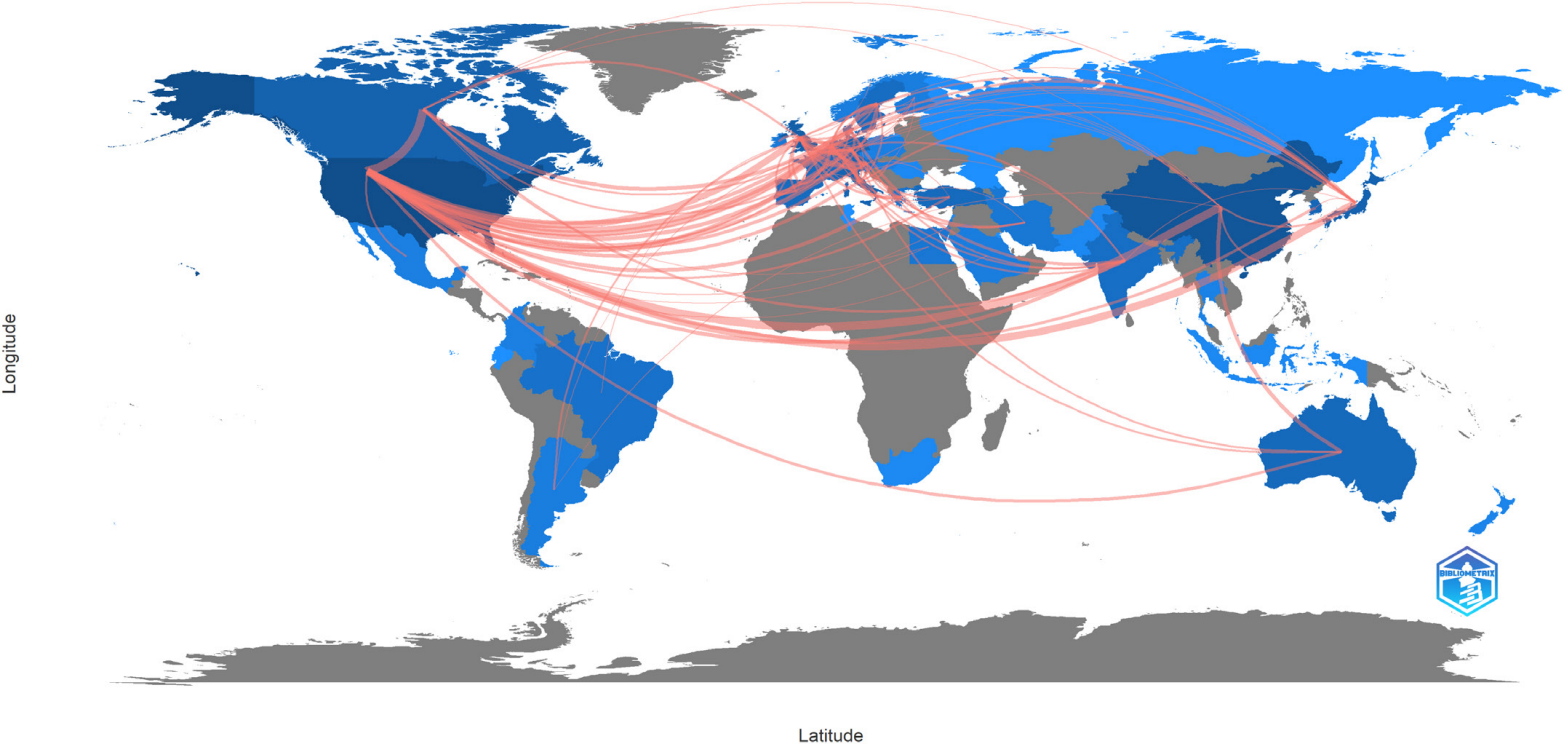
Country collaboration map.

**Figure 10.**
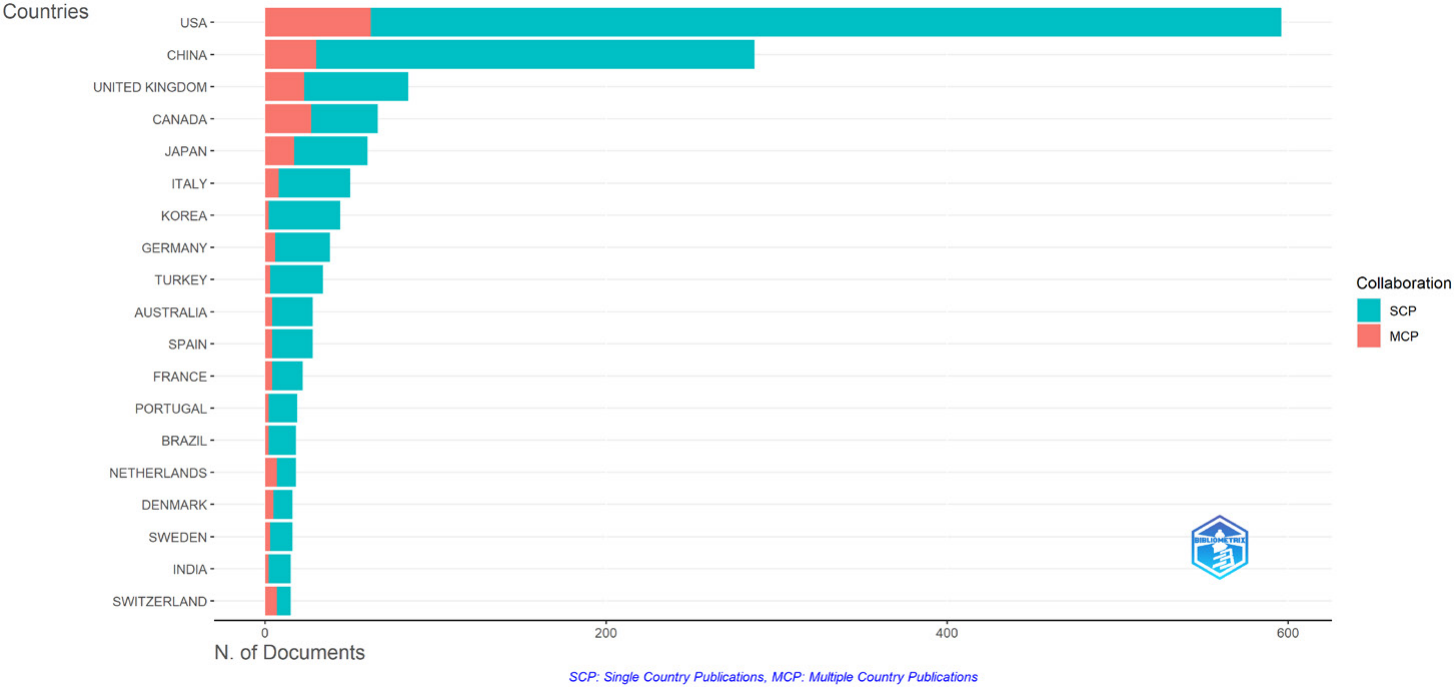
Corresponding author’s countries.

The most relevant countries reported were the USA and China, which were also reported to be the top most cited countries. Figure 8 shows each country’s scientific production and Figure 9 indicates the country’s collaboration map. As shown in Figure 10, the corresponding authors of the included studies were mostly from the USA and China.

**Figure 11.**
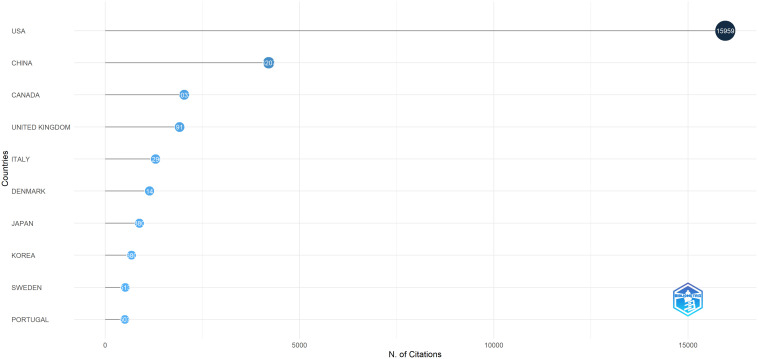
Most cited countries.

**Figure 12.**
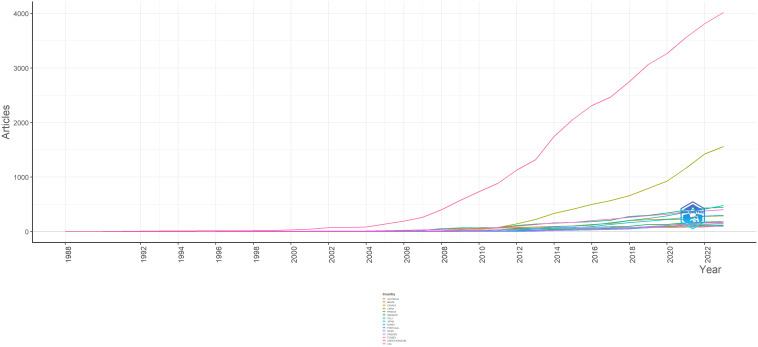
Country production over time.

The most cited articles originated from the USA, while China is in second place. Figure 11 ranks the countries based on their citations and Figure 12 shows the growth in production over time between countries.

### Co-Word Evaluation

**Figure 13.**
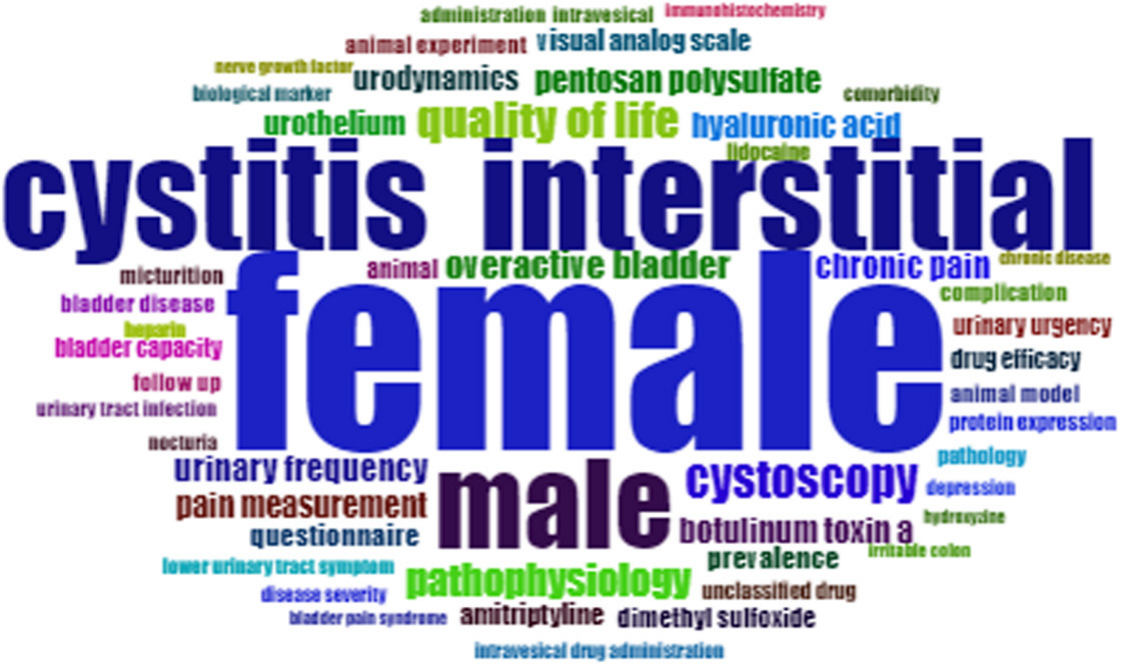
Word cloud of relevant words used by studies.

**Figure 14.**
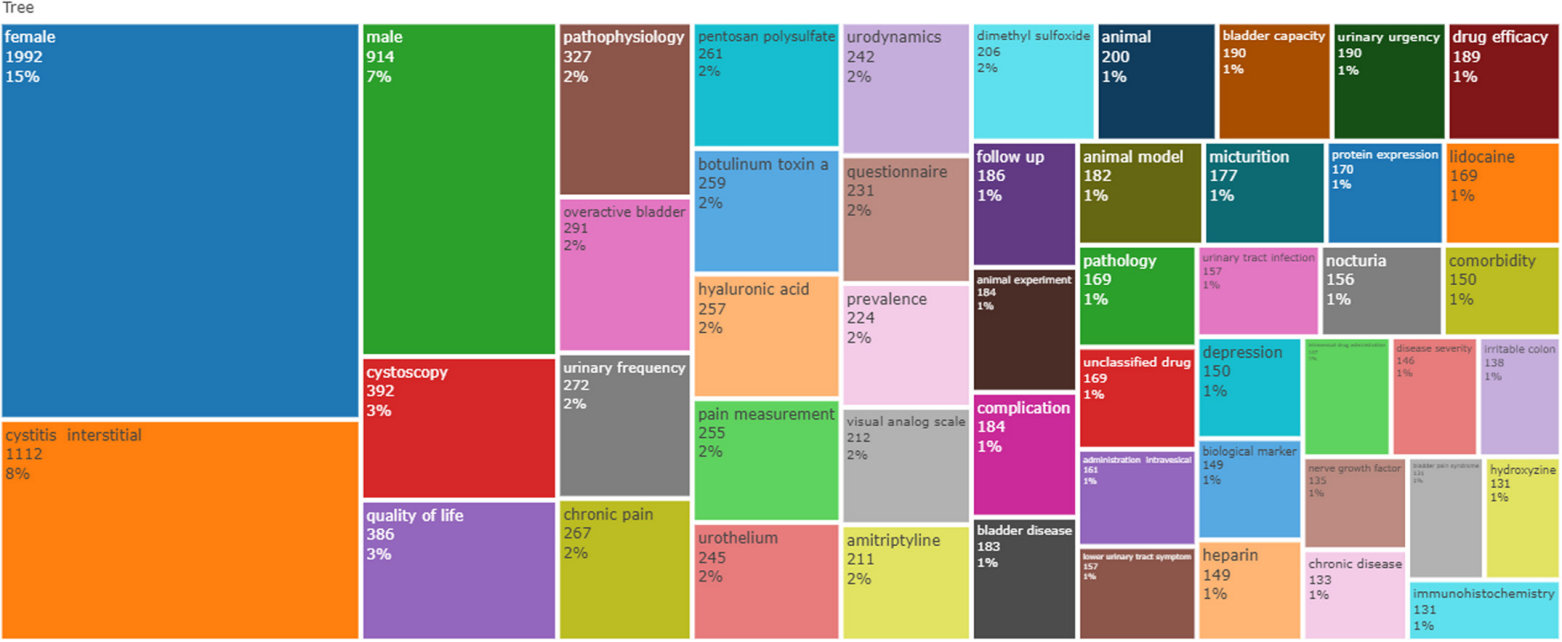
Keyword tree map.

**Figure 15.**
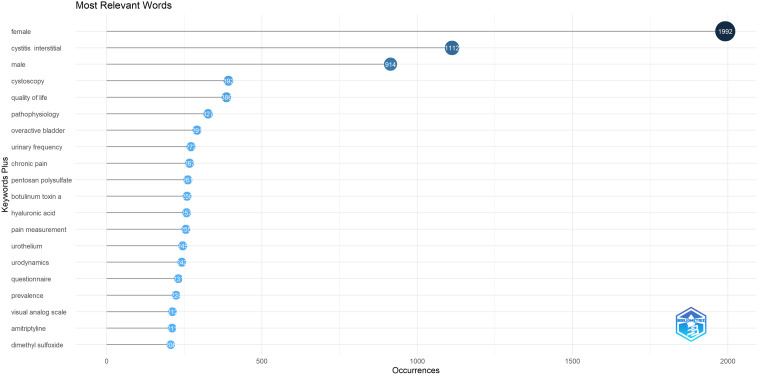
Most relevant words.

**Supplementary Figure 1.**
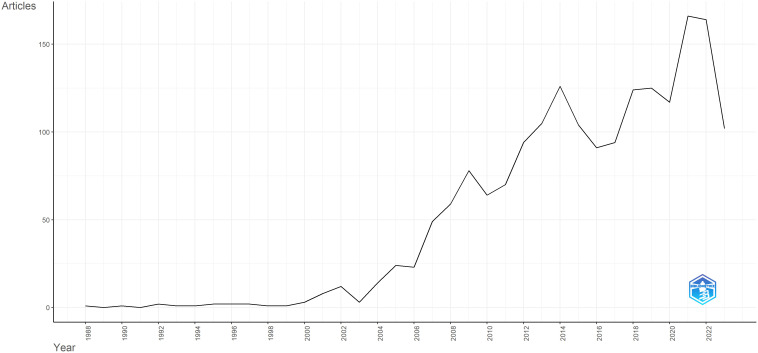
Trend topics over time.

This section focuses on the co-occurrence of keywords in research topics and shows their interactions.^12^ Figure 13 presents a word cloud highlighting the primary focus areas and Figure 14 demonstrates a keyword tree map. As illustrated in Figure 15, the most relevant words are “female,” “interstitial cystitis,” “human,” “cystitis interstitial,” “male,” and “cystalgia.” Supplementary Figure 1, provided in supplementary file 1, shows the trend topics in IC/BPS according to each year. With this analysis, monitoring the evolution of research topics in IC/BPS is possible. Genome-wide association and Cannabidiols are currently the hot topics of IC/BPS.

**Supplementary Figure 2.**
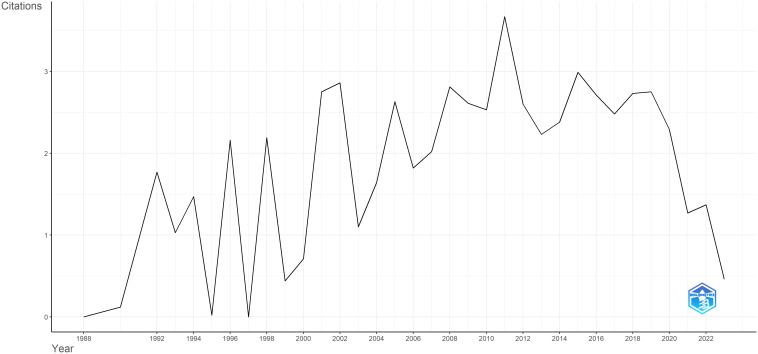
Thematic map of interstitial cystitis/bladder pain syndrome studies.

Supplementary Figure 2, provided in supplementary file 1, comprises 4 parts: motor themes, niche themes, emerging or declining themes, and basic themes. Basic themes are important topics about which insufficient knowledge exists and are suggested for future research focus. Motor themes are important subjects with sufficient data, and niche themes are unimportant subjects with too much research in those areas. Emerging and declining themes are topics of low importance and a low number of surveys. These topics are generally not suggested. The analysis highlights key trends and themes in the field of IC/BPS, particularly focusing on the thematic categorization of research topics. Here’s a structured summary and interpretation of the information:

### Motor Themes

- The identified motor themes are crucial topics that are well-addressed in current research. Keywords associated with these themes include:
- Pentosan Polysulfate: Often used for bladder pain syndrome/interstitial cystitis.
- Botulinum Toxin A: Relevant for treating an overactive bladder.
- Hyaluronic Acid: Used in bladder instillations.
- Amitriptyline: An antidepressant sometimes used for chronic pain management.
- Dimethyl Sulfoxide: Utilized for its anti-inflammatory properties.
- Lidocaine: A local anesthetic often used in urological procedures.
- Cystoscopy: A procedure critical to diagnosing various urological conditions.
- Overactive Bladder: A common condition that significantly impacts the quality of life.

## Emergency or Declining Themes

In contrast, the emergency or declining themes represent areas that are currently perceived as less important or less prioritized within the field. This group includes keywords such as:

- Animal Studies: While foundational, there may be a shift toward human studies or clinical applications.
- Protein Expression, Pathology, Urothelium: These terms suggest a focus on basic science that may not directly translate into clinical practice or lack immediate clinical relevance.
- Rat Models and Pathophysiology: While essential for understanding disease mechanisms, these areas may not attract as much attention compared to more immediate clinical applications.

**Supplementary Figure 3.**
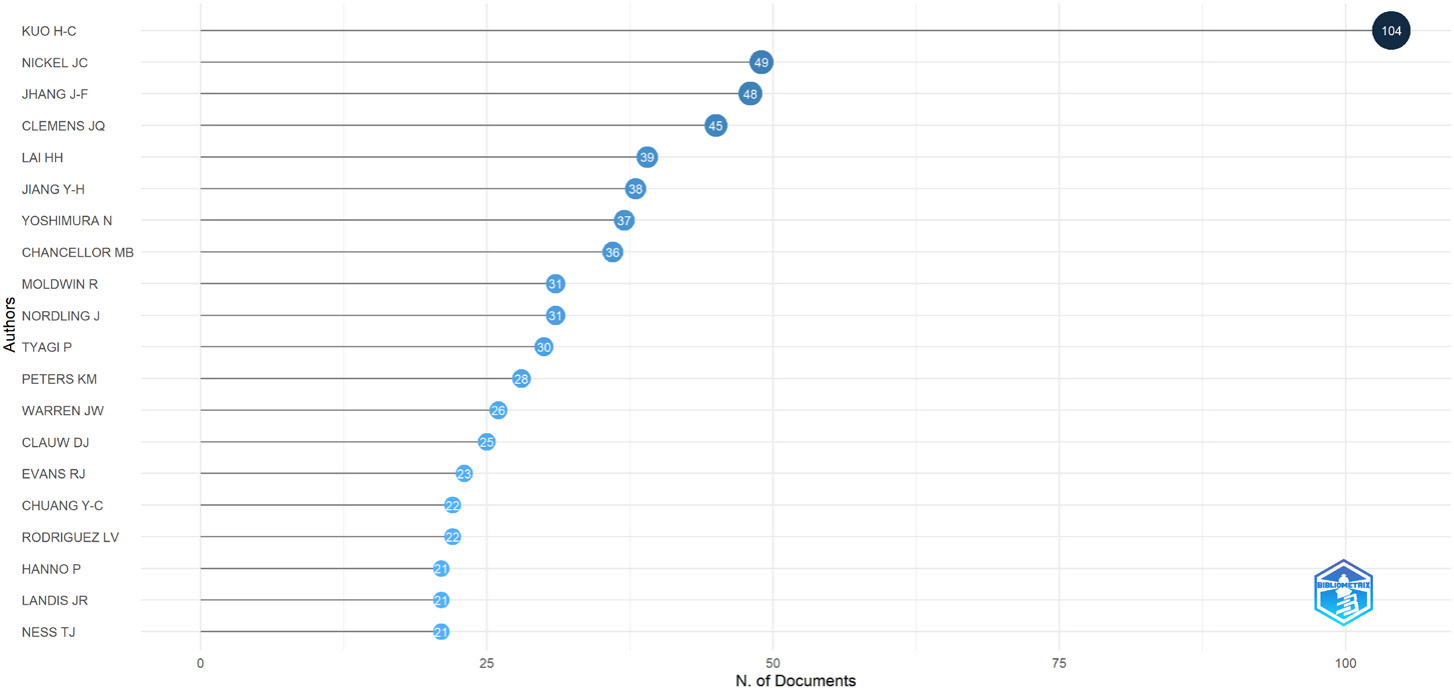
Thematic evolution.

Supplementary Figure 3 illustrates the thematic evolution.

**Supplementary Figure 4.**
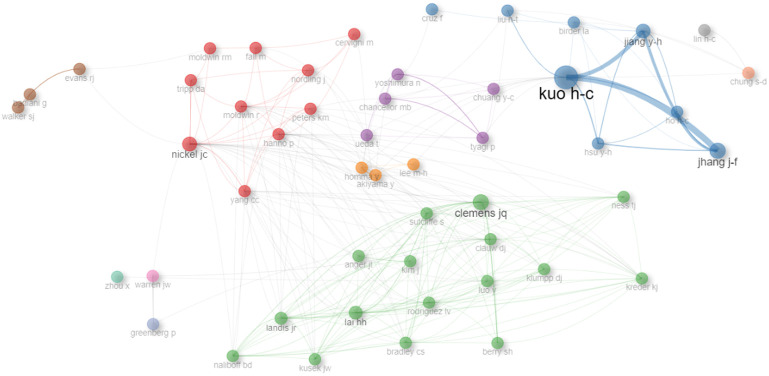
Topics clustered by authors coupling.

Supplementary Figure 4, provided in supplementary file 1, clusters topics by authors’ coupling in 3 categories (green, blue, and red).

### Co-citation Evaluation

Different subgroup analyses in this section are author, document, and journal co-citation analyses.

**Supplementary Figure 5.**
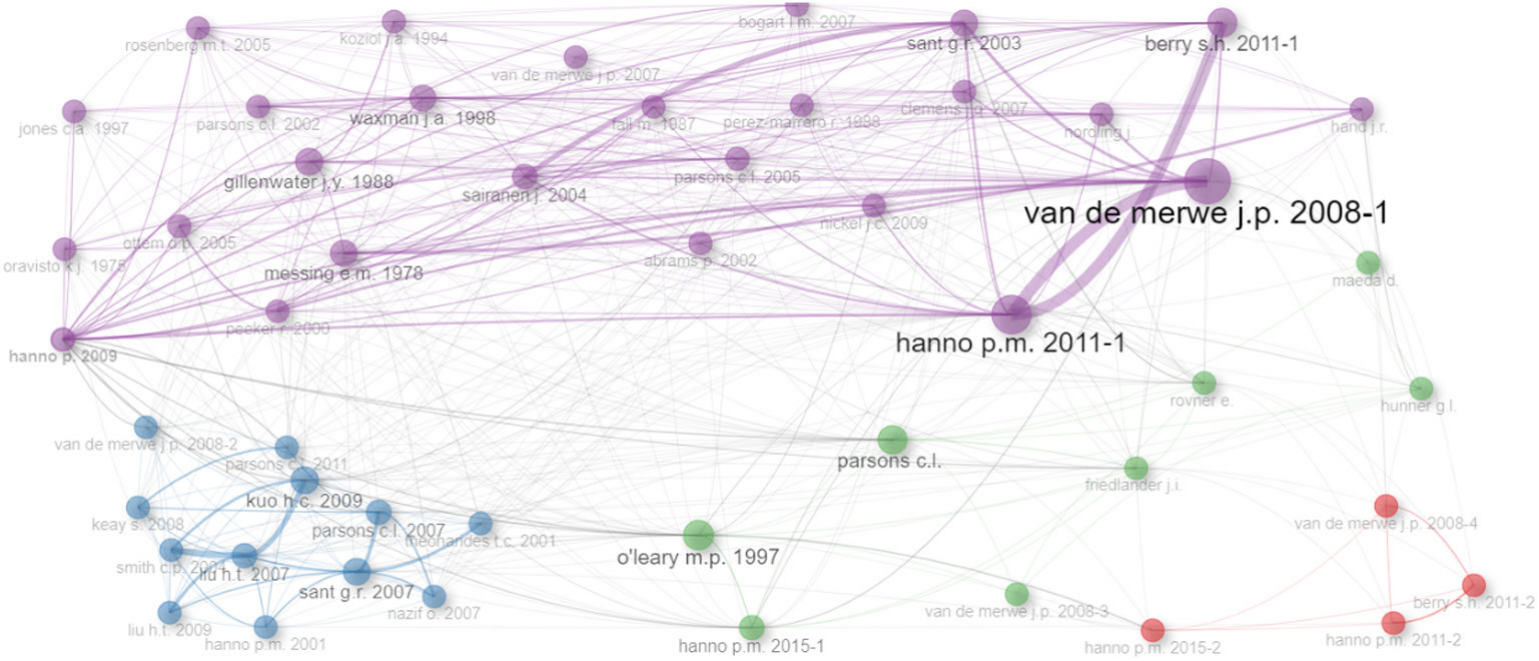
Most relevant affiliations.

**Supplementary Figure 6.**
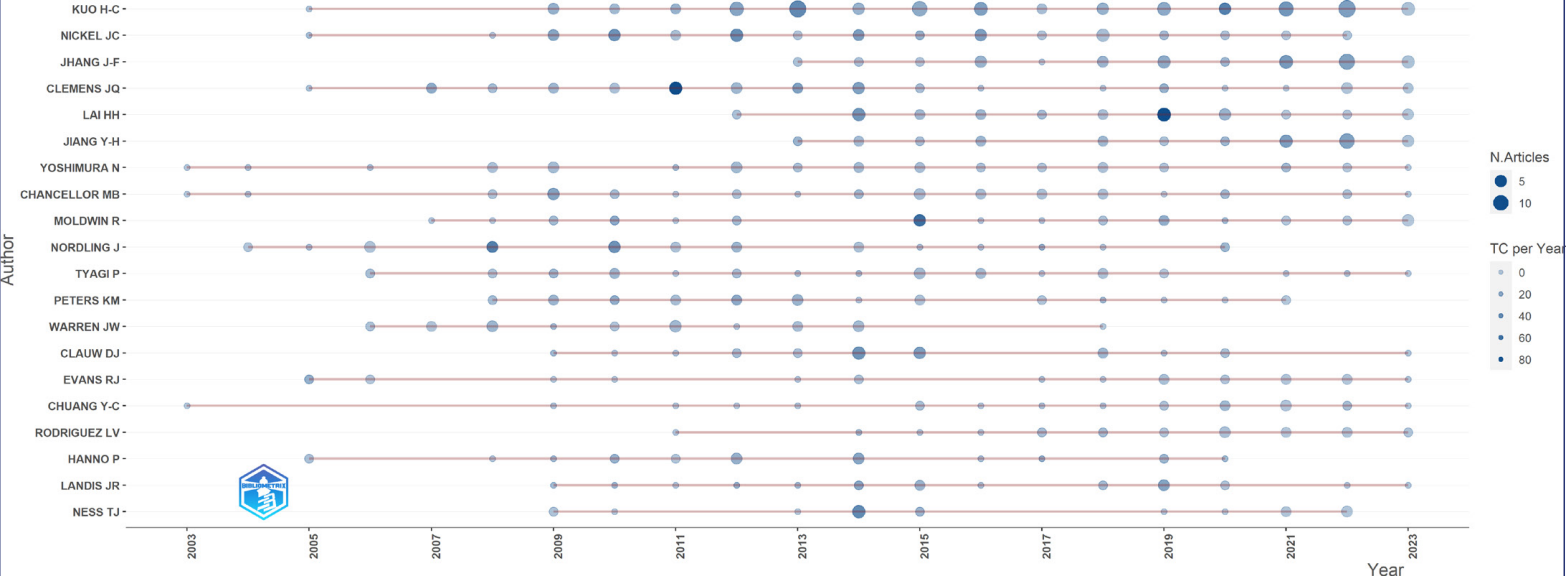
Affiliations’ production over time.

The most relevant affiliations were TZU CHI University, with 154 articles, and the University of Pittsburgh, with 121 articles. Supplementary Figure 5, provided in supplementary file 1, represents the most relevant affiliations, and Supplementary Figure 6, provided in supplementary file 1, is a demonstration of each affiliation’s production over time.

**Supplementary Figure 7.**
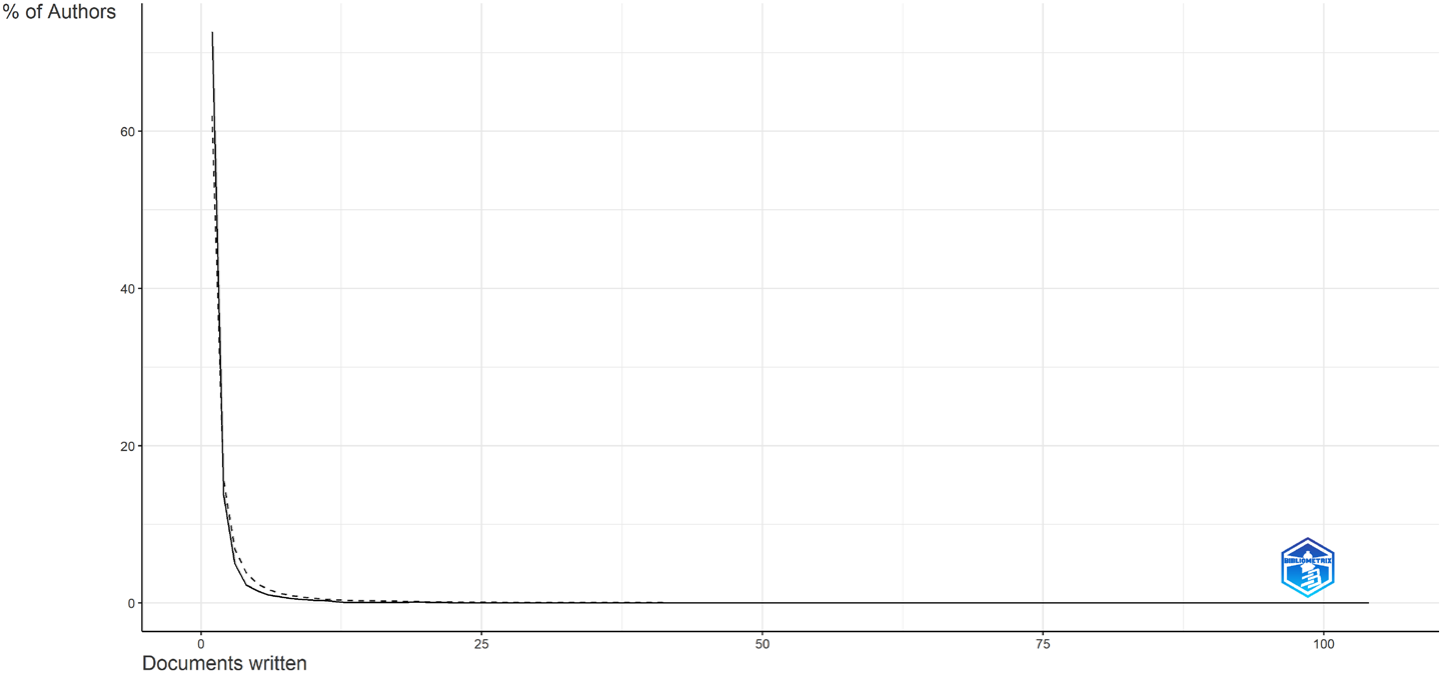
Most relevant sources.

**Supplementary Figure 8.**
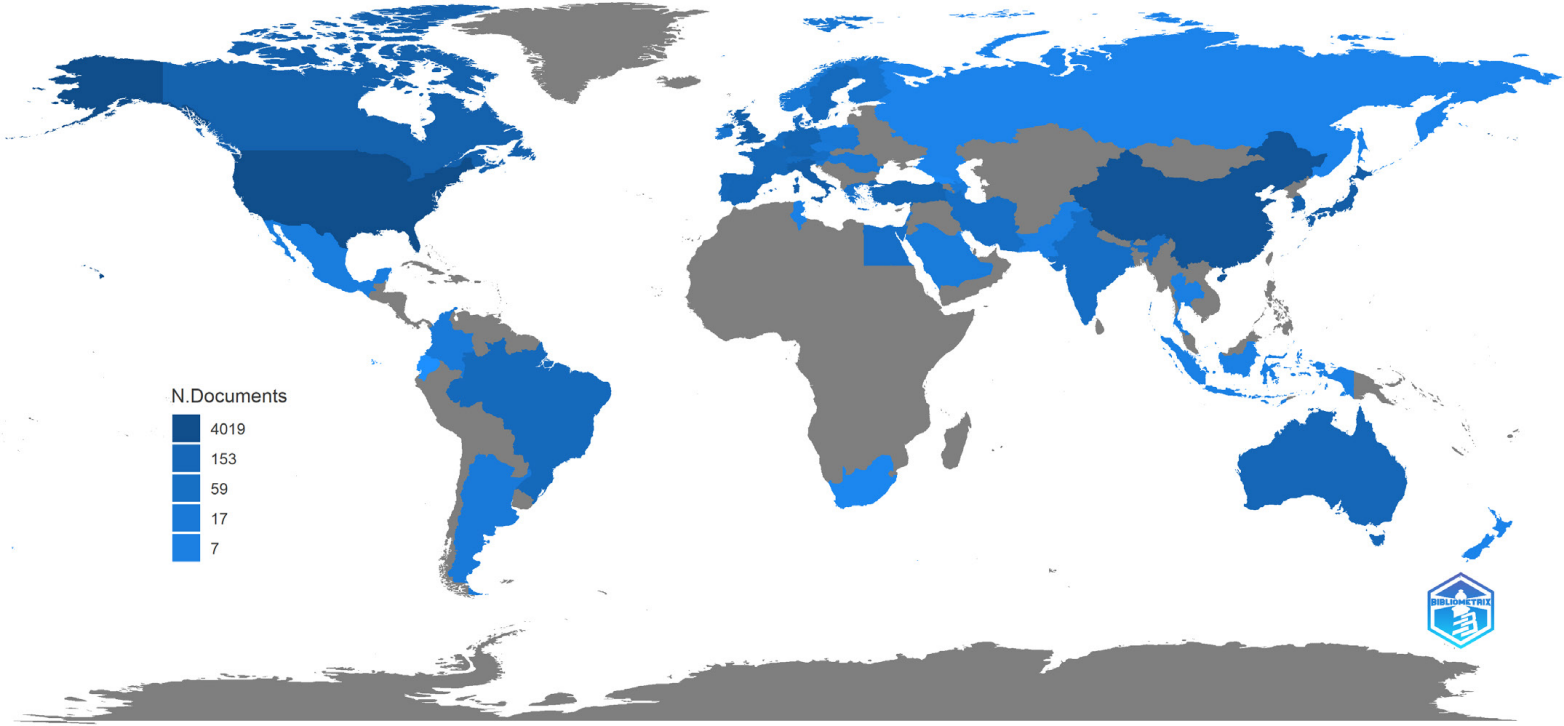
Sources production over time.

The most relevant sources were the *Journal of Urology* and the *Journal of Neurourology and Urodynamics*, each containing 125 documents. Supplementary Figures 7 and 8, both provided in supplementary file 1, present the most relevant sources in this field and their production over the years.

**Supplementary Figure 9.**
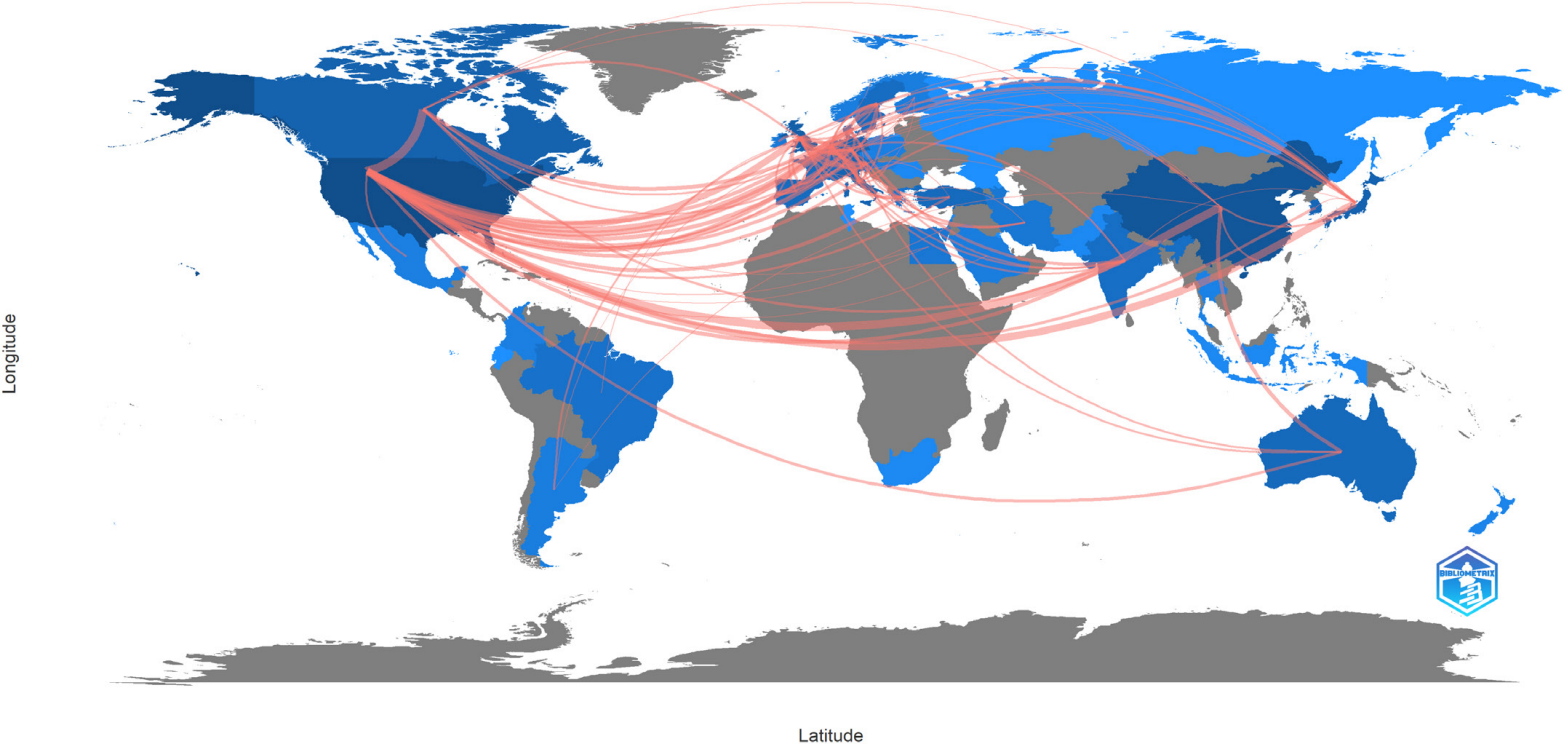
Reference publication year spectroscopy.

**Supplementary Figure 10.**
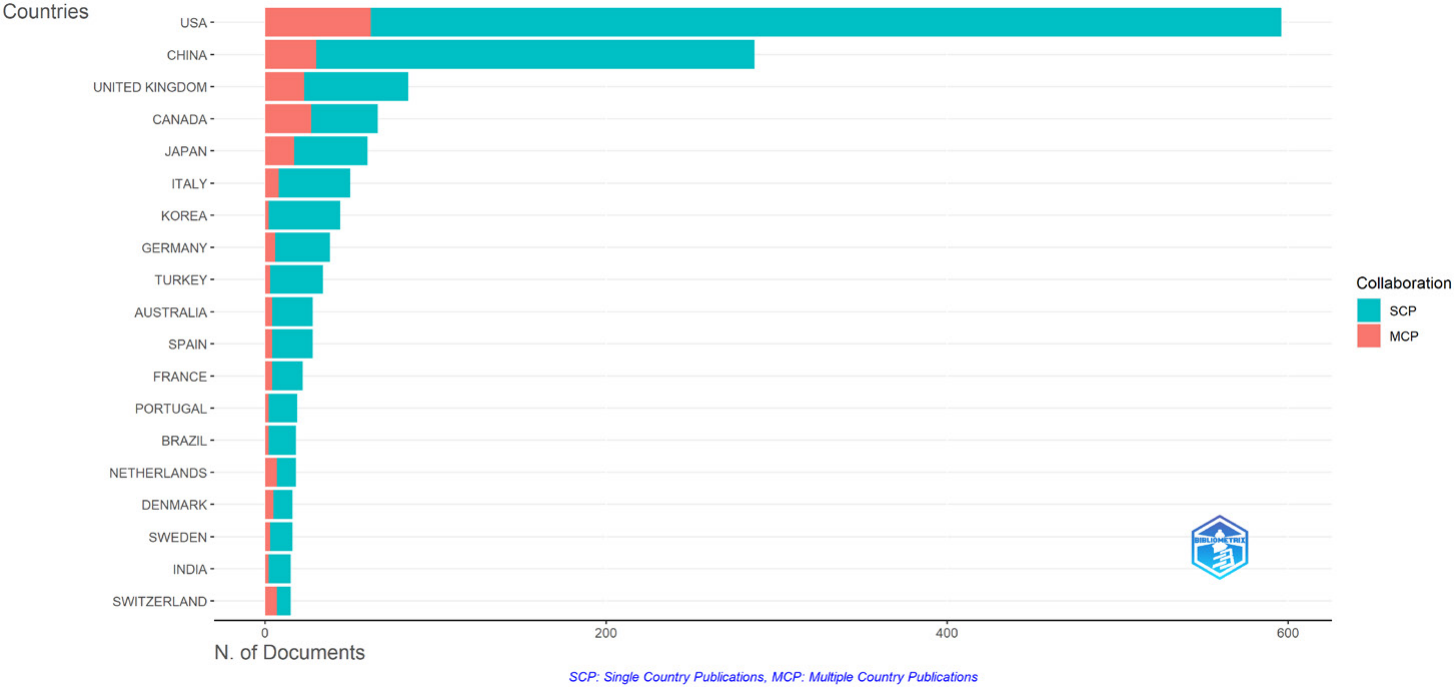
The three-field plot of the relation between keywords, countries, and the institutions’ affiliations.

**Supplementary Figure 11.**
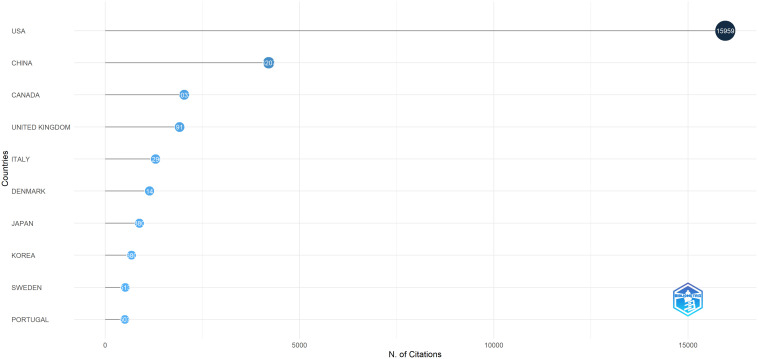
Core sources by Bradford’s law.

Supplementary Figure 9, provided in supplementary file 1, illustrates the rate of scientific production from 1985 to 2018. Supplementary Figure 10, provided in supplementary file 1, shows a 3-field plot of the relation between keywords, countries, and the institutions’ affiliations. Supplementary Figure 11, provided in supplementary file 1, represents the core sources in IC/BPS by Bradford’s law.

**Supplementary Figure 12.**
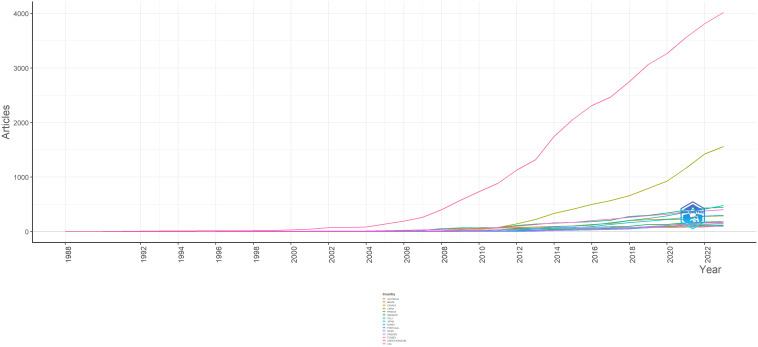
Most globally cited documents.

Van De Merwe et al (2008) published the most globally cited article in the *European Urology journal*. Hanno et al (2011), Hanno et al (2015), and Berry et al (2011) had the second to fourth ranks in this section, and all were published in the *Journal of Urology*. Supplementary Figure 12, provided in supplementary file 1, shows the most cited documents globally.

The co-occurrence network is presented in Supplementary Figure 3. According to the results, female and interstitial cystitis comprised the most co-occurrence network.

## Discussion

The importance of studying IC/BPS lies in its significant impact on the quality of life of patients, the economic burden it poses, and the need for effective treatments. IC/BPS can lead to a decrease in work productivity, emotional changes, sleep disturbances, and sexual dysfunction. The condition is often associated with comorbid conditions such as depression, anxiety, and sleep disorders, which can further exacerbate its effects on quality of life. Therefore, novel research approaches, patient-reported outcomes, and healthcare policy are all critical components of studying IC/BPS and improving patient outcomes.^13^

This study provided insights into research trends in IC/BPS. Diagnostic, basic science, and therapeutic studies were prominent, with notable contributions from authors like Kuo H.C. and Nickel J.C. Collaboration networks and co-citation analyses revealed significant partnerships and influential authors. The USA and China led scientific production, with notable growth observed in the past few years. Prominent keywords such as “female” and “genome-wide association” emerged, indicating notable areas of focus. Key affiliations and journals were identified, with seminal articles garnering global recognition.

The analysis of relevant terms within the discourse surrounding IC/BPS sheds light on prevailing research themes and emerging areas of interest. Keywords such as “female,” “interstitial cystitis,” “human,” “cystitis interstitial,” “male,” and “cystalgia” underscore the importance of understanding the epidemiological and clinical dimensions of IC/BPS, particularly with regard to gender-specific manifestations and patient populations. The inclusion of “genome-wide association” and “cannabidiols” as hot topics within IC/BPS research reflects the evolving landscape of therapeutic interventions and molecular investigations in the field. For instance, in a study by Estrella et al,^14^ variants in the genes SIX5, ATP2C1, and ATP2A2 were identified, providing insights into certain aspects of the IC/BPS phenotype in affected individuals. Therefore, genome-wide association studies offer insights into the genetic underpinnings of IC/BPS susceptibility and variability, potentially paving the way for personalized treatment approaches. Concurrently, the exploration of cannabidiols as a novel therapeutic method signifies a growing interest in harnessing the therapeutic properties of cannabinoids for managing IC/BPS symptoms, including pain and inflammation. Kuret et al^15^ offer new insights into the therapeutic possibilities of cannabidiol by highlighting its modulation of PPARγ, Nrf2, and NFκB signaling pathways, potentially opening avenues for its application in IC/BPS treatment. By identifying these prominent terms and topics, researchers can delineate current research priorities, inform future investigations, and advance understanding and management strategies for IC/BPS.

The insights gleaned from the scientometric analysis of research on IC/BPS, particularly regarding the prominence of the USA and China as leading contributors to the scientific discourse, underscore the global significance of this condition. The extensive scientific production from these countries reflects their active engagement and investment in IC/BPS research. Moreover, the collaboration map highlights the importance of international collaboration in advancing knowledge in this field, suggesting opportunities for cross-border partnerships to further enhance research outcomes and address the complexities of IC/BPS comprehensively. The dominance of the USA in citation ranking, along with the notable contributions from China and Canada, emphasizes the impact of research originating from these nations on shaping the understanding and management of IC/BPS.^16^ Furthermore, the growth in research production observed in these countries over time signals a growing awareness and interest in IC/BPS globally. This underscores the increasing recognition of IC/BPS as a significant healthcare challenge and emphasizes the importance of continued research efforts to improve diagnosis, treatment, and patient outcomes worldwide.

Despite significant advances in understanding IC/BPS, there remains a substantial gap between research findings and practical application in clinical settings. These gaps include pathophysiology, understanding the symptoms, detecting abnormalities inside or outside the bladder, verifying that these abnormalities cause the symptoms, and therapeutic methods. A recent study by Tornic et al^17^ highlights various underlying mechanisms, such as impaired urothelial barrier function, changes in urothelial factors and cytokines, chronic inflammation, vascular lesions, neurogenic inflammation, and processes in the central nervous system leading to central sensitization. Based on these mechanisms, IC/BPS can be categorized into clusters according to clinical phenotypes, which aids in clinical decision-making and treatment. Moreover, one of the critical areas where this gap is evident is in the role of the microbiome in urological diseases, including IC/BPS. While emerging research suggests that the microbiome could significantly impact lower urinary tract dysfunctions such as overactive bladder syndrome, chronic prostatitis, interstitial cystitis, incontinence, prostate cancer, and urolithiasis, translating these findings into practical treatments has been slow.^18^

The diverse nature and multiple causes of IC/BPS may explain why clinical trials involving broad patient groups without specific subtypes have mostly failed to find new treatments in the last decade. Therefore, identifying specific subtypes of IC/BPS, such as those focused on bladder issues or beyond, including identifying Hunner or non-Hunner lesions via cystoscopy, is essential for future treatment. Since Hunner-lesion IC has distinct inflammatory characteristics and tissue damage, it has been suggested in recent presentations at international conferences—such as the International Consultation on IC in Japan—that it be treated differently from non-Hunner IC/BPS. Nevertheless, traditional cystoscopy can miss atypical or small lesions, and there are no established standards for detecting Hunner lesions other than the normal ones. Furthermore, it is necessary to confirm that the identified reported mucosal lesions are actually producing bladder discomfort in order to diagnose the bladder-focused variety of IC/BPS.^19^

The International Consultation on Incontinence IC/BPS group reported in 2023 that IC and BPS are primarily the same since there is no precise definition that distinguishes IC from BPS. In the absence of additional medical conditions, IC/BPS is commonly characterized as persistent pelvic pain, discomfort, or pressure related to the bladder, along with at least one other urinary symptom, such as frequent or continuous urine urgency. According to the Consultation, individuals who fit this description and have a Hunner lesion have to be treated as if they have Hunner lesion disease. To establish whether the condition is Hunner lesion disease or IC/BPS, early cystoscopy is essential. Since most patients do not respond well to only 1 treatment over time, the course of treatment should be customized to address each patient’s particular set of symptoms, frequently necessitating a multidisciplinary approach.^20^ This precision in diagnosis is essential to tailor treatments effectively and improve patient outcomes. However, the implementation of such diagnostic advancements in routine clinical practice remains inconsistent.

The lifestyle and behavioral adjustments patients with IC/BPS implement to control their symptoms were investigated in a study by Lin et al.^21^ The study, which was conducted from 2018 to 2019, included cystoscopic evidence as well as patients who had experienced lower urinary tract symptoms such as suprapubic pain for longer than 6 weeks. A self-created questionnaire regarding the participants’ living and working conditions, clothes for work, eating habits, and personal habits was filled out by the patients. According to the study, individuals with IC/BPS frequently alter their lifestyles and behaviors in an effort to manage their symptoms. Furthermore, according to a recent study by McKernan et al,^22^ individuals with IC/BPS may benefit from tailored psychological therapies that address sexual dysfunction and intimacy-related concerns, as well as pain management, emotion regulation, and communication skills.

The integration of multidisciplinary approaches, incorporating insights from IC/BPS research, urothelial biology, and personalized medicine, is essential. This integrated approach can provide a more comprehensive treatment plan that addresses the varied symptoms and underlying causes of IC/BPS. To achieve this, ongoing education and training for healthcare providers, as well as increased collaboration between researchers and clinicians, are vital.

### Limitations of Scopus Database

The primary limitation of the scientometric analysis was the reliance on a single Scopus search. While prior research^23^ indicates that Scopus, the largest abstract and citation database of peer-reviewed literature, provides datasets that accurately reflect various fields due to its extensive indexing of high-impact factor journals, this approach still has inherent constraints. Scopus offers a comprehensive overview of global research across science, technology, medicine, social sciences, and the arts and humanities, with advanced tools for tracking, analyzing, and visualizing research outputs. However, by limiting the analysis to journals indexed in Scopus, some relevant papers may have been missed inadvertently, potentially overlooking important insights and developments in IC/BPS research.

### Implications for Future Studies

The findings of this study provide several important implications for future research in the field of IC/BPS:

- Increased Scientific Production: The significant rise in scientific production post-2000 suggests a growing interest and investment in IC/BPS research. Future studies could explore the factors contributing to this increase, such as advancements in research methodologies, funding opportunities, and rising awareness of the condition among healthcare professionals and patients.
- Collaboration Networks: The analysis of collaboration networks indicates a concentration of productivity among certain authors and institutions, particularly in the USA and China. Future research should consider fostering international collaborations to enhance knowledge sharing and innovation. Identifying and engaging with leading authors and institutions could lead to more impactful studies and comprehensive reviews in the field.
- Focus on Co-Word Evaluation: The co-word analysis highlights key areas of interest, such as “female,” “interstitial cystitis,” and “genome-wide association.” Future studies should prioritize these emerging topics, especially those identified as basic themes with insufficient knowledge, like IC/BPS pathophysiology. This could lead to breakthroughs in understanding the underlying mechanisms of the condition.
- Exploration of Declining Themes: The identification of declining themes suggests a shift in research focus. Future studies should critically assess the relevance of these topics and consider redirecting resources toward more promising areas of inquiry that align with current clinical needs and gaps in knowledge.
- Diversity in Research Topics: The keyword trends indicate a need for broader research beyond the currently popular themes. Investigating less-explored areas could yield valuable insights and contribute to a more holistic understanding of IC/BPS.
- Geographic Trends: Given the dominance of the USA and China in both publication volume and citations, future studies should explore the global landscape of IC/BPS research. This could include comparative analyses of research outputs across countries and regions and identifying unique challenges or advancements in different healthcare settings.
- Methodological Innovations: As noted in the findings, the average number of citations per document is relatively high, suggesting that impactful studies are being published. Future research should continue to adopt innovative methodologies, including interdisciplinary approaches that integrate genetics, pharmacology, and patient-reported outcomes to enhance the robustness of findings.
- Longitudinal Studies: Given the evolving nature of research topics, longitudinal studies that track changes over time will be crucial. These will help identify emerging trends and shifts in research priorities, providing insights into how the field is adapting to new evidence and societal needs.
- Engagement with Emerging Technologies: The highlighted interest in genome-wide associations and cannabinoids suggests potential avenues for future exploration. Researchers should engage with cutting-edge technologies and therapeutic approaches, investigating their efficacy and safety in managing IC/BPS.

The analysis underscores the dynamic nature of urology research, revealing both areas of robust interest and those that may require renewed focus. By aligning research efforts with motor themes, stakeholders can enhance clinical outcomes and address pressing issues within the field. Conversely, recognizing declining themes may prompt a reevaluation of resource allocation to ensure a balanced approach between basic science and clinical application.

- Research Focus Shift: The emphasis on motor themes indicates a shift towards practical applications and treatments that directly affect patient care, such as pharmacological interventions and minimally invasive procedures. This reflects a growing demand for research that translates into effective clinical practices.
- Declining Interest in Basic Science: The emergence of declining themes suggests a potential gap in funding or interest for basic science research within the IC/BPS field. This could lead to a lack of foundational knowledge necessary for future advancements.
- Clinical Relevance: The prominence of certain keywords highlights the importance of patient-centered approaches in IC/BPS research. Topics that directly impact treatment efficacy and patient outcomes are gaining traction.

### Recommendations for Researchers, Policymakers, and Stakeholders

For researchers, it is essential to foster interdisciplinary collaboration across various fields such as urology, pain management, and genetics. This collaboration can lead to comprehensive studies that address the multifactorial nature of IC/BPS. Researchers should prioritize underexplored areas identified in previous studies, particularly those focusing on pathophysiology and psychosocial aspects. Longitudinal studies are crucial for tracking changes in symptoms and treatment outcomes over time, providing insights into disease progression. Engaging patients in the research process through participatory methods can ensure that studies are relevant and focused on improving patient outcomes. Additionally, leveraging genomic and biomarker research can facilitate the exploration of personalized treatment options, including emerging therapies like cannabinoids. Establishing global research networks will allow for the sharing of knowledge and resources, particularly with institutions in countries that are increasing their research output.

For clinicians, continuous education is vital to staying informed about the latest findings and treatment modalities for IC/BPS. Adopting a holistic treatment approach that includes specialists from various fields—such as pain management, physical therapy, and mental health—can address the complex needs of patients more effectively. Utilizing standardized diagnostic criteria and assessment tools will improve the accuracy of diagnoses and treatment plans. Comprehensive patient education is critical; clinicians should provide information about IC/BPS, potential treatment options, lifestyle modifications, and coping strategies to empower patients in managing their condition. Furthermore, clinicians should encourage patient participation in ongoing clinical trials to contribute to advancing the understanding and treatment of IC/BPS.

For policymakers, increasing funding for IC/BPS research through grants and public health initiatives is essential to stimulate innovation and attract new researchers to the field. Launching public awareness campaigns can help raise awareness about IC/BPS among healthcare providers and the general public, leading to improved early diagnosis and reduced stigma associated with the condition. Supporting multidisciplinary care models will ensure that patients have access to a range of specialists involved in their care. Policymakers should also encourage the establishment of national databases to collect data on IC/BPS prevalence, treatment outcomes, and patient experiences, which can facilitate better research and policy decisions. Advocacy for patient rights is crucial; policymakers must ensure equitable access to treatment options for all patients with IC/BPS, regardless of socioeconomic status or geographic location. Lastly, facilitating partnerships between researchers, healthcare providers, patient advocacy groups, and industry stakeholders will create a cohesive strategy for advancing care and research in IC/BPS.

By implementing these recommendations, stakeholders can work together to bridge existing gaps in IC/BPS research and care, ultimately improving outcomes for patients affected by this challenging condition.

## Conclusion

In conclusion, while research on the microbiome and other emerging fields holds promise for improving the understanding and treatment of IC/BPS, significant efforts are needed to translate these findings into practical, standardized, and effective clinical practices. Addressing these gaps will require a concerted effort to update diagnostic criteria, adopt personalized treatment strategies, and ensure that healthcare providers are well-informed about the latest research developments. This analysis illustrates a clear distinction between themes actively shaping the future of IC/BPS and those needing reevaluation. The emphasis on motor themes indicates a shift towards practical, patient-centered research, while the recognition of emerging or declining themes highlights potential gaps in foundational studies that could inform future advancements. By focusing on these motor themes, researchers and clinicians can enhance patient outcomes and tackle the most pressing challenges in urology today.

## Figures and Tables

**Supplementary Figure 13.**
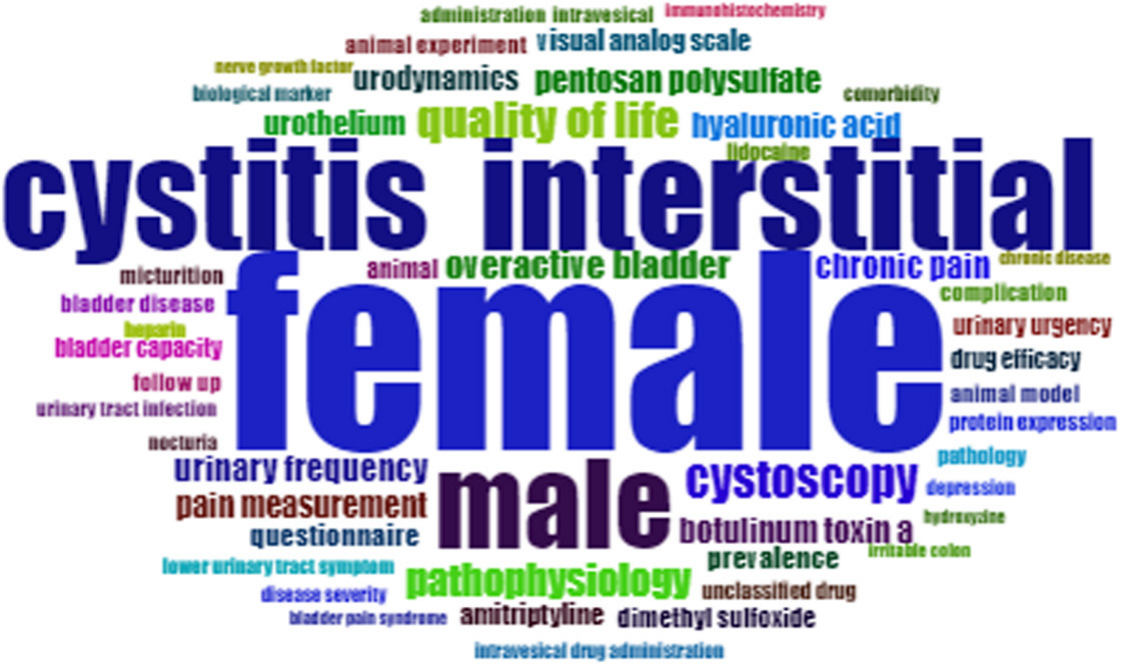
Co-occurrence network.

**Table 1.** Main Information of Included Studies

Description	Results
Timespan	1988:2023
Sources	477
Documents	1833
Annual growth rate %	14.13
Document average age	7.47
Average citations per doc	20.75
References	52815
**Document Contents**	
Keywords plus (ID)	8554
Author’s keywords (DE)	2420
**Authors**	
Authors	5276
Authors of single-authored docs	117
**Authors Collaboration**	
Single-authored docs	154
Co-Authors per doc	5.36
International co-authorships	14.68
**Document Types**	
Article	1326
conference paper	29
Review	478

## Data Availability

The data supporting this study’s findings are available from the corresponding authors, Sakineh Hajebrahimi and Hanieh Salehi-Pourmehr, upon reasonable request.
