# Supplementary material for: Gap of Research to Practice in IC/BPS: A Scientometric Study of Available Evidence
Source: Urol Res Pract. 2025 Apr 4;50(6):332–42. doi: 10.5152/tud.2025.24086 (PMC12015755; doi:10.5152/tud.2025.24086)
Supplement: Supplementary Material [file supplementary_material.pdf]

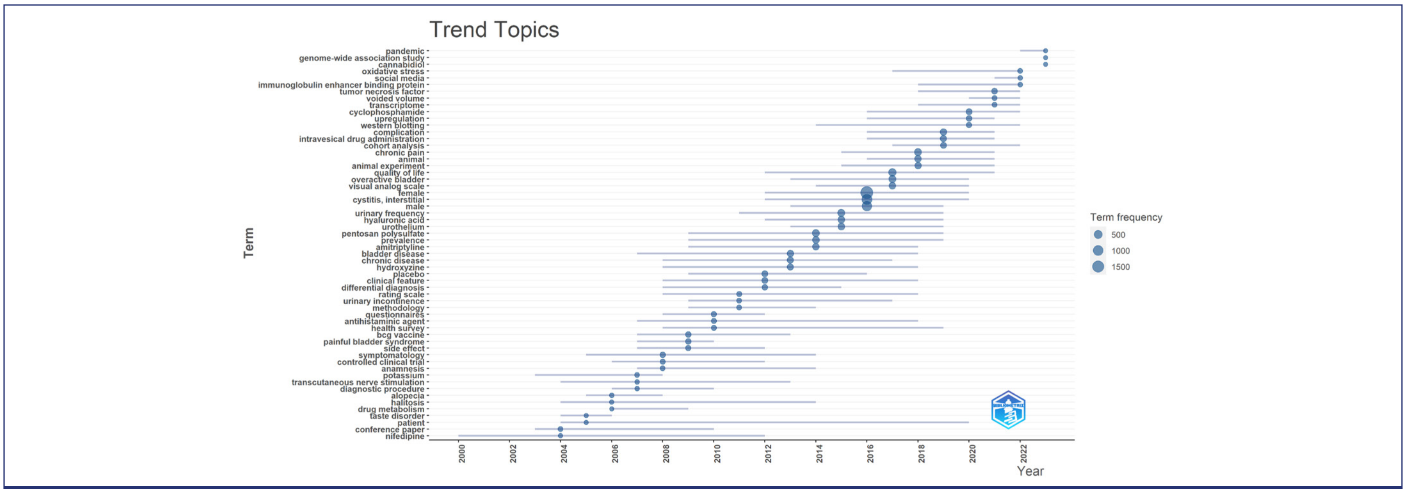

Supplementary Figure 1. Trend topics over time.

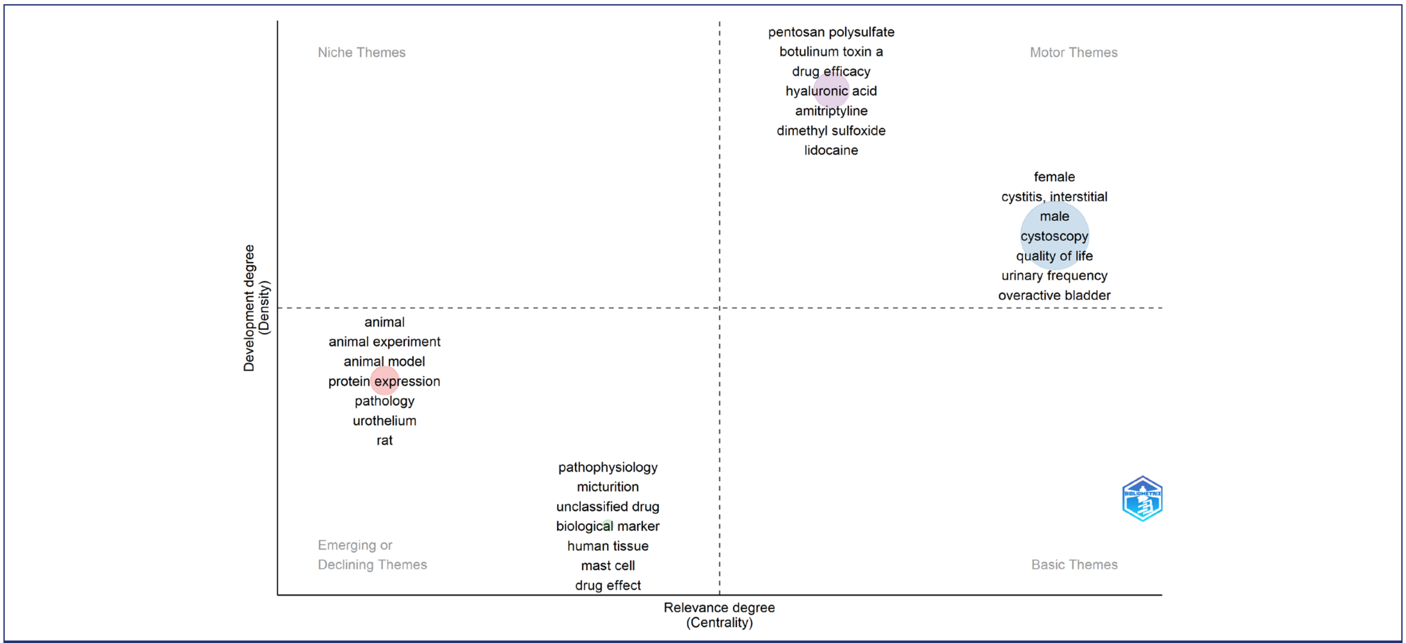

Supplementary Figure 2. Thematic map of interstitial cystitis/bladder pain syndrome studies.

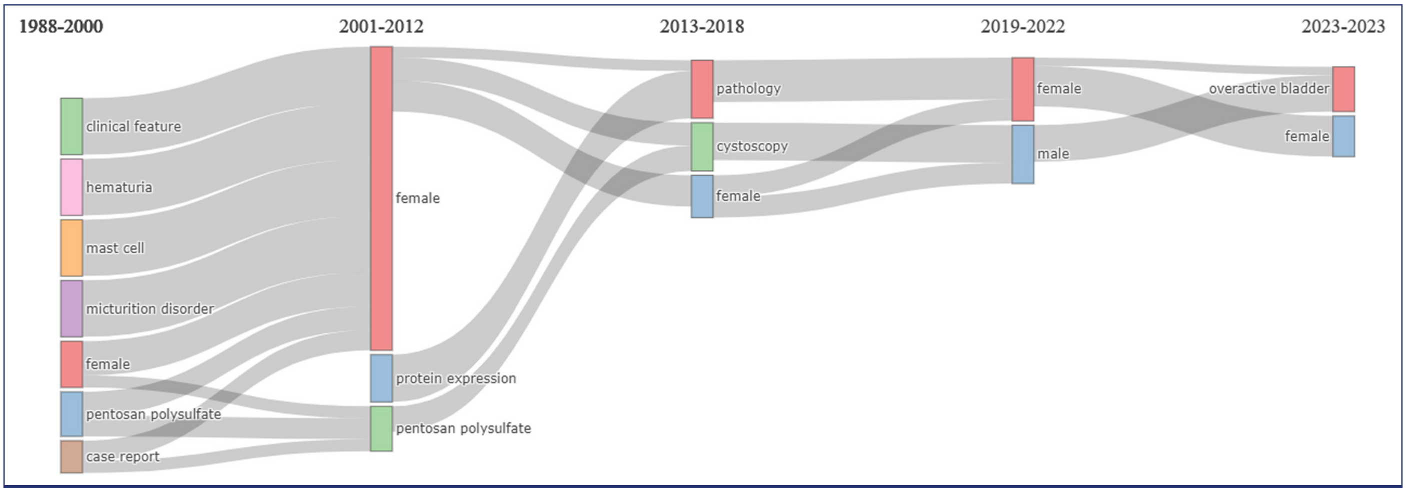

Supplementary Figure 3. Thematic evolution.

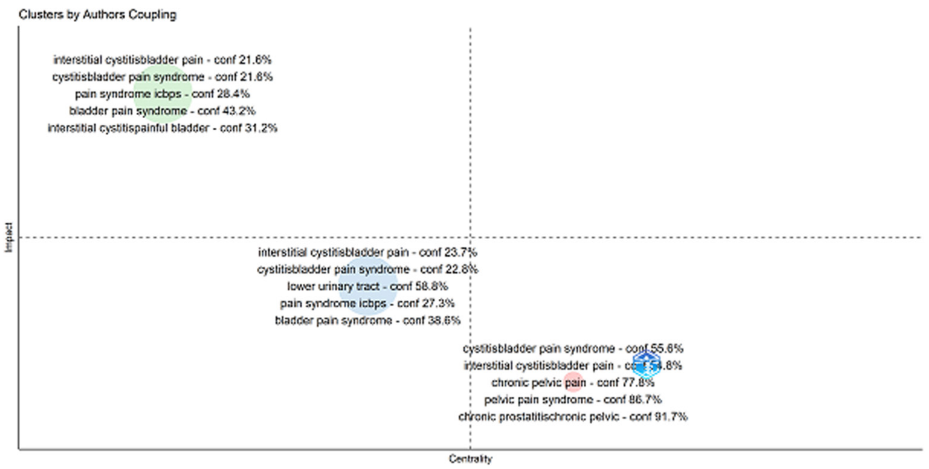

Supplementary Figure 4. Topics clustered by authors coupling.

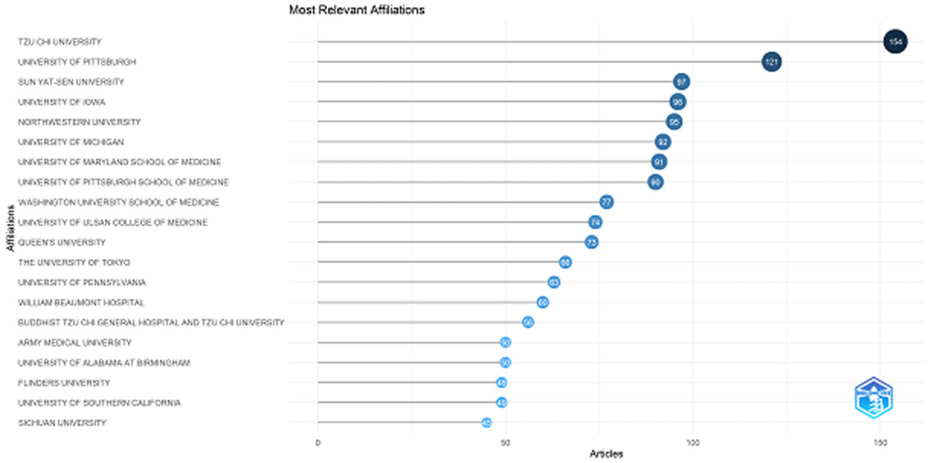

Supplementary Figure 5. Most relevant affiliations.

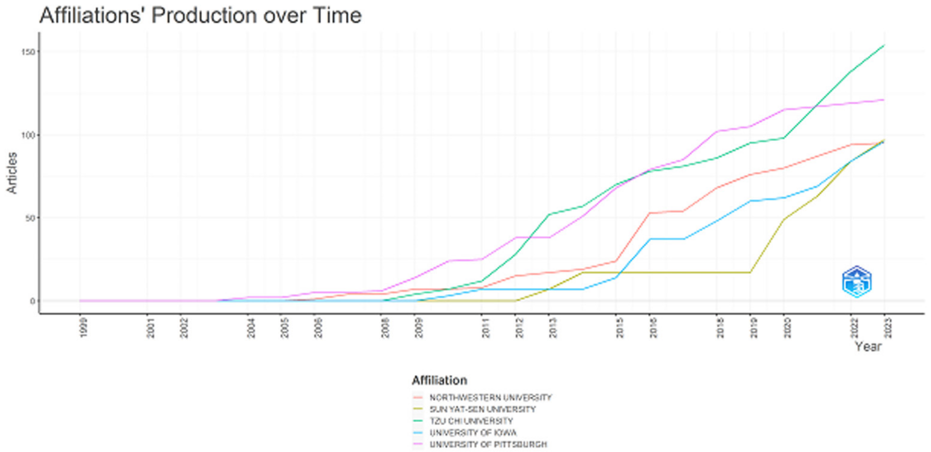

Supplementary Figure 6. Affiliations' production over time.

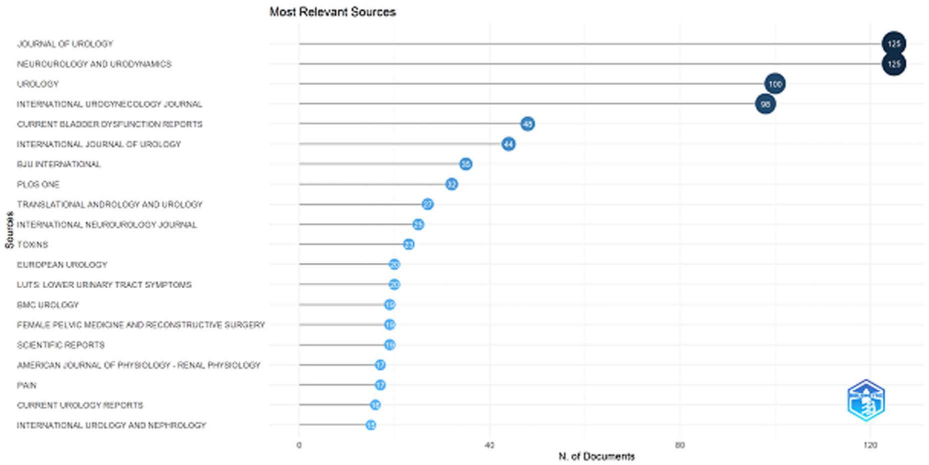

Supplementary Figure 7. Most relevant sources.

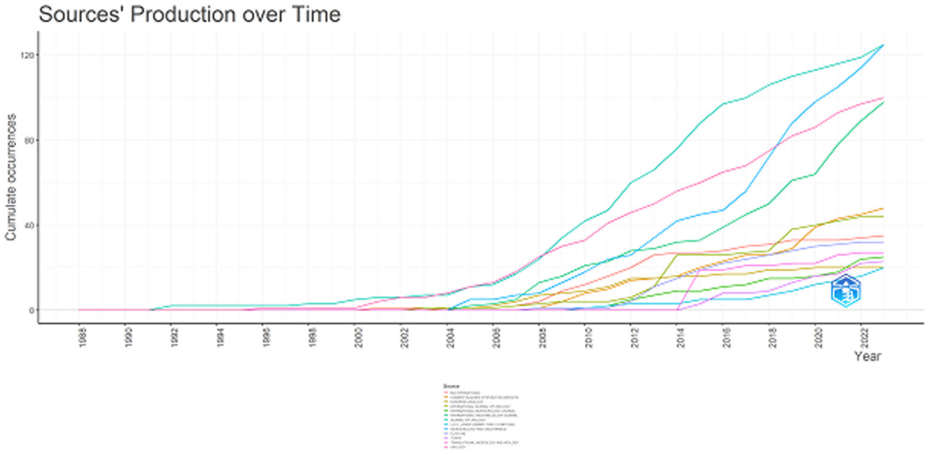

Supplementary Figure 8. Sources production over time.

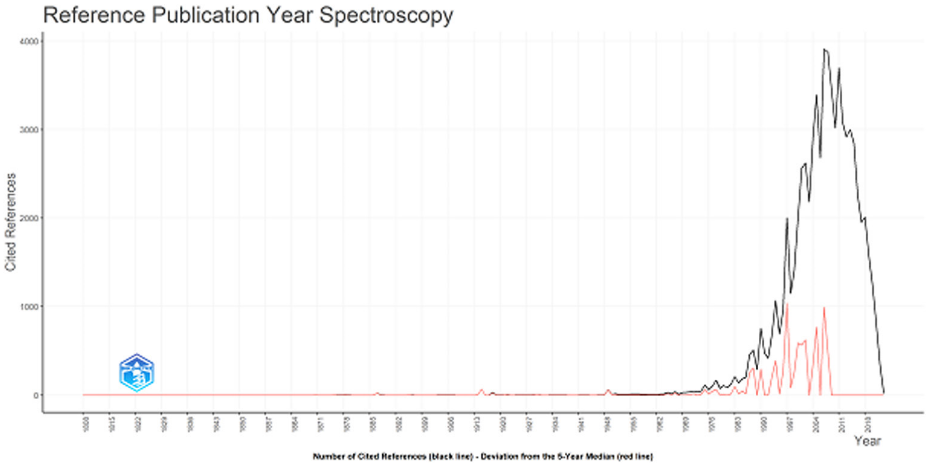

Supplementary Figure 9. Reference publication year spectroscopy.

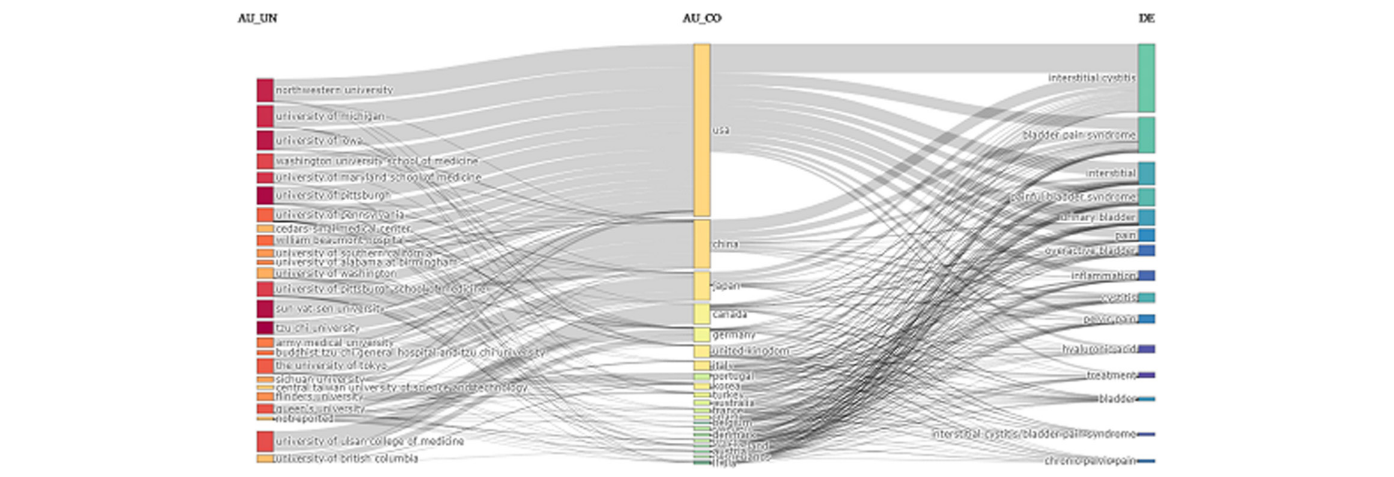

Supplementary Figure 10. The three-field plot of the relation between keywords, countries, and the institutions' affiliations.

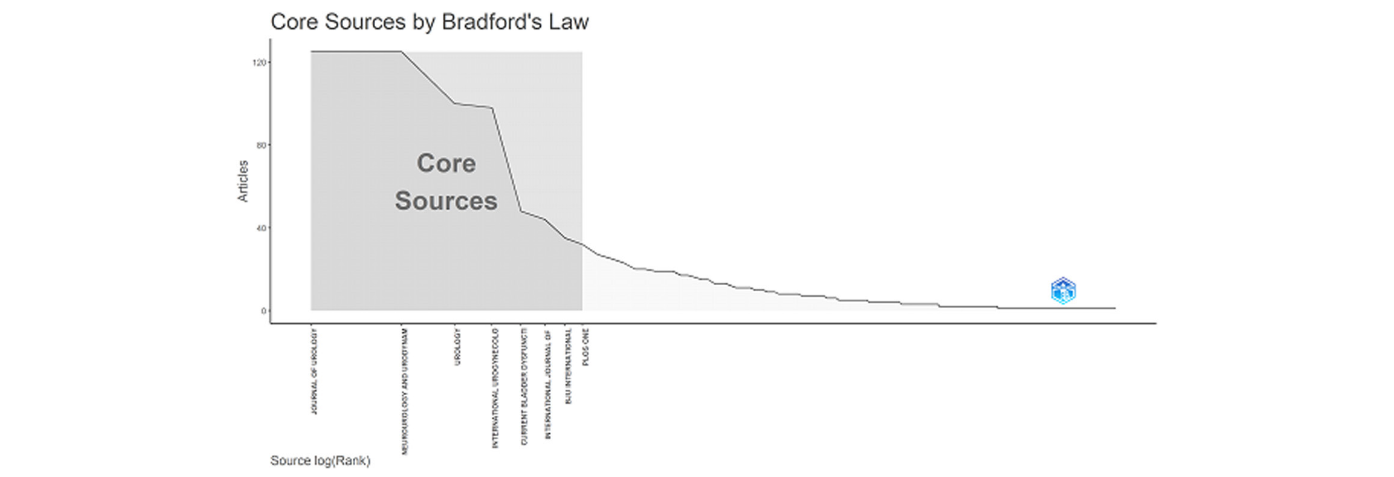

Supplementary Figure 11. Core sources by Bradford's law.

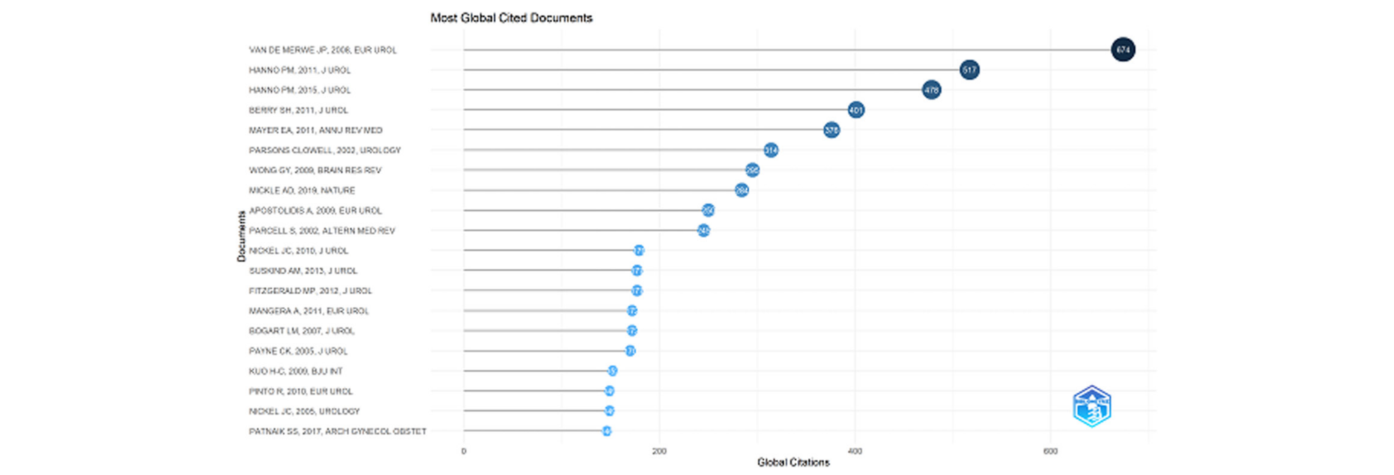

Supplementary Figure 12. Most globally cited documents.

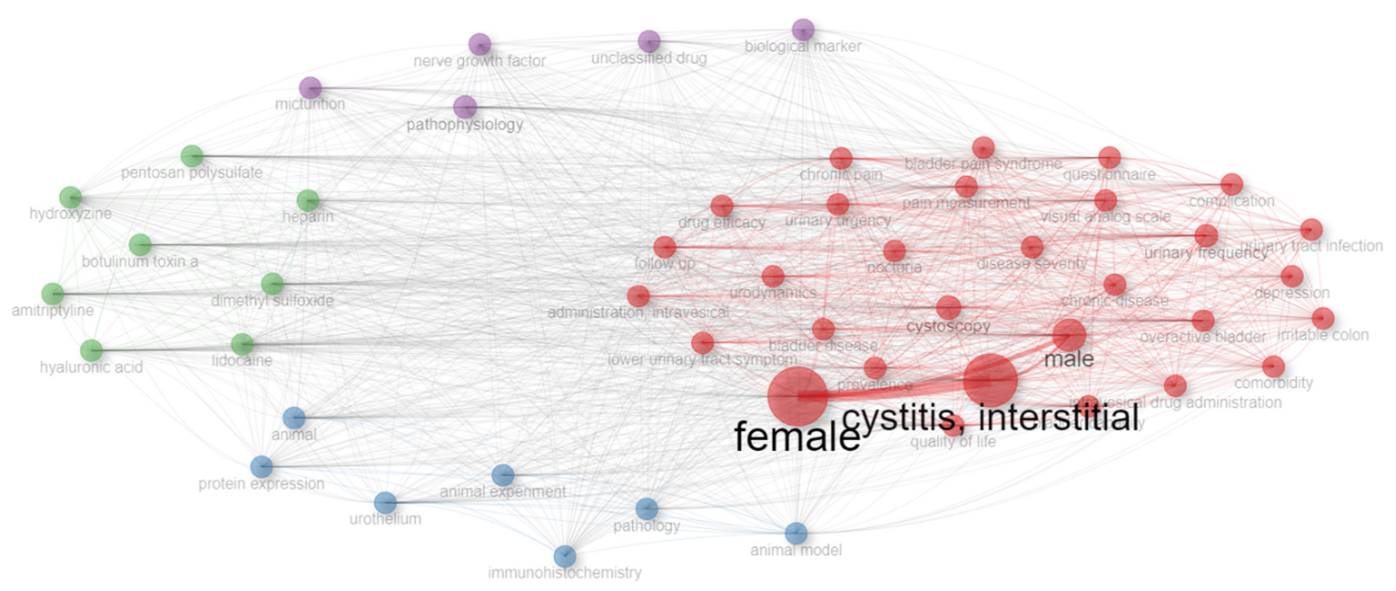

Supplementary Figure 13. Co-occurrence network.
